# IGFBP2 enhances adipogenic differentiation potentials of mesenchymal stem cells from Wharton's jelly of the umbilical cord *via* JNK and Akt signaling pathways

**DOI:** 10.1371/journal.pone.0184182

**Published:** 2017-08-31

**Authors:** Yuejun Wang, Yunsong Liu, Zhipeng Fan, Dayong Liu, Fu Wang, Yongsheng Zhou

**Affiliations:** 1 Department of Prosthodontics, Peking University School and Hospital of Stomatology, Beijing, China; 2 Laboratory of Molecular Signaling and Stem Cells Therapy, Beijing Key Laboratory of Tooth Regeneration and Function Reconstruction, Capital Medical University School of Stomatology, Beijing, China; 3 Department of Oral Basic Science, School of Stomatology, Dalian Medical University, Liaoning, China; 4 National Engineering Laboratory for Digital and Material Technology of Stomatology, Beijing, China; University of Miami School of Medicine, UNITED STATES

## Abstract

Mesenchymal stem cell (MSC)-mediated tissue engineering represents a promising strategy to address adipose tissue defects. MSCs derived from Wharton’s jelly of the umbilical cord (WJCMSCs) may serve as an ideal source for adipose tissue engineering due to their abundance, safety profile, and accessibility. How to activate the directed differentiation potentials of WJCMSCs is the core point for their clinical applications. A thorough investigation of mechanisms involved in WJCMSC adipogenic differentiation is necessary to support their application in adipose tissue engineering and address shortcomings. Previous study showed, compared with periodontal ligament stem cells (PDLSCs), WJCMSCs had a weakened adipogenic differentiation potentials and lower expression of insulin-like growth factor binding protein 2 (*IGFBP2*). IGFBP2 may be involved in the adipogenesis of MSCs. Generally, IGFBP2 is involved in regulating biological activity of insulin-like growth factors, however, its functions in human MSCs are unclear. Here, we found *IGFBP2* expression was upregulated upon adipogenic induction, and that *IGFBP2* enhanced adipogenic differentiation of WJCMSCs and BMSCs. Moreover, IGFBP2 increased phosphorylation of c-Jun N-terminal kinase (p-JNK) and p-Akt, and activated JNK or Akt signaling significantly promoted adipogenic differentiation of MSCs. Furthermore, inhibitor-mediated blockage of either JNK or Akt signaling dramatically reduced *IGFBP2*-mediated adipogenic differentiation. And the JNK inhibitor, SP600125 markedly blocked *IGFBP2*-mediated Akt activation. Moreover, *IGFBP2* was negatively regulated by *BCOR*, which inhibited adipogenic differentiation of WJCMSCs. Overall, our results reveal a new function of *IGFBP2*, providing a novel insight into the mechanism of adipogenic differentiation and identifying a potential target mediator for improving adipose tissue engineering based on WJCMSCs.

## Introduction

Adipose tissue plays an essential role in the maintenance of organ contours, energy storage, metabolic balance, and immune regulation through exocrine hormones and fat cell factors. Adipose tissue defects due to tumor resection, trauma, or hereditary and congenital diseases usually lead to loss of fat tissue, poor appearance and local disordered regulatory function. A major clinical challenge is that traditional treatments such as prosthetic appliance, plastic and reconstructive surgery and fat grafting do not effectively restore adipose tissue. Adipose tissue engineering and cell-based therapies represent novel and promising approaches for regenerating adipose tissue.

MSCs have been isolated from various tissues, including bone marrow, adipose tissue, vascular tissue, dental tissue, craniofacial tissue, and umbilical cord [1–7]. With their convenient isolation, low immunogenicity, and ability to transdifferentiate, MSCs are considered a promising therapeutic approach for tissue regeneration [1,4,5,8,9]. WJCMSCs, which are isolated from neonatal umbilical cord tissue, are a plentiful, cost-effective, and biologically safe source of stem cells and show the significant potential for regenerative medicine [9–12]. As studied dental-derived stem cell population, PDLSCs which own the higher stemness features and preferable multi-differentiation properties, are the ideal seeding cells for tissue regeneration [8,13,14]. However, some researches suggested that compared with PDLSCs, unmodified WJCMSCs with their weaker stemness features might not be ideal seed cells for tissue regeneration [8,15]. A crucial issue for WJCMSCs-mediated adipose tissue regeneration is how to activate adipogenic differentiation and enhance regenerative ability.

As the essential member of insulin-like growth factor (IGF) axis, insulin-like growth factor binding proteins (IGFBPs) are homologs with high structural similarity but distinct functionalities. They all have similar N-terminal and C-terminal domains connected by a variable linker region [16]. IGFBPs assume a key regulatory role in many cellular processes, including proliferation, migration, differentiation, and survival. IGFBPs also play an essential role in the processes of growth, development, and tissue metabolism [17]. In the IGF axis, IGFs play influential roles in the function of IGFBPs [18,19]. Several studies indicated that IGF1 was an essential regulator of adipogenic differentiation. IGF1 was shown to upregulate phosphorylation of cAMP response element-binding protein (CREB) through the PI3K/Akt pathway, and then activated CREB increased the expression of *PPARγ2*, which was a crucial factor in adipogenesis through regulating specific gene expression [20–22]. IGFBP2 was the predominant binding protein secreted by differentiating white preadipocytes. In chickens, the *IGFBP2* gene could be a candidate locus or linked to a major gene associated with abdominal fat weight and percentage of abdominal fat [23,24]. Our previous research showed that, compared with dental derived stem cells, WJCMSCs exhibited decreased adipogenic differentiation potential as well as downregulated expression of *IGFBP2* [15]. These findings suggested the possible involvement of *IGFBP2* in the regulation of adipogenic differentiation in MSCs.

Many events facilitate the commitment of MSC adipogenic differentiation, including the coordination of a complex network of transcription factors, co-factors, and pathway signaling intermediates. The extracellular regulated protein kinases (ERK), p38, and JNK MAPK family are a group of serine/threonine kinases that transduce extracellular signals to intracellular targets, involving a series of protein kinase cascades and long-term response that play a crucial role in regulating cell differentiation [25–27]. Many researches focused on the effect of the MAPK family on adipogenic differentiation. Sale et al. found that ERK1 and ERK2 were required for differentiation of 3T3-L1 fibroblasts to adipocytes [28]. And inhibited ERK pathway by specific inhibitor could restrain adipocyte differentiation ability [29]. In addition, ERK activity was essential for the expressions of the *PPARγ* and *C/EBP* [30,31]. Moreover, cells isolated from *Erk*^*-/-*^ mouse showed impaired adipogenesis capability [32]. It was previously reported that the JNK pathway also regulated adipogenesis differentiation of MSCs [33]. JNK could phosphorylate PPARγ2 by oxidized low-density lipoprotein [34]. Yet, using SP600125, a specific JNK inhibitor, could increase the expressions of *CEBPα/β* and *PPARγ2*, and stimulate adipogenesis of hASCs in a dose-dependent manner [35]. Moreover, the drug for preventing osteoporosis, alendronate, inhibited adipogenic differentiation by ERK and JNK pathway in BMSCs [36]. As for the role of p38 in adipogenic differentiation, some studies showed that using p38 inhibitors could block adipocyte differentiation [37,38]. In addition, the Akt signaling pathway was also essential for inducing *PPARγ*. Akt activity sustained the adipogenic differentiation of ASCs. *Akt* knockout mice showed impaired adipogenesis [39–41]. Importantly, *IGFBP2* could activate multiple MAPK pathways. *IGFBP2/Integrin5* interaction promoted glioma cells migration through JNK activation [42]. Exogenous IGFBP2 induced proliferation and activated the ERK pathway in NIH-OVCAR3 cells, and also promoted proliferation in rat growth plate chondrocytes via MAPK/ERK pathway [43]. In addition, the expression of *IGFBP2* was positively regulated by PI3K/Akt pathway, and the Akt signal transduction was impaired in *Igfbp2* ^-/-^ mouse cells [44]. However, it is still unknown the effect of *IGFBP2* on MAPK and Akt pathways during adipogenic differentiation of WJCMSCs. Based on the available information, we hypothesize that *IGFBP2* affects the function of MSCs, but its function and mechanism remain unclear. Here, we investigate the effects and underlying mechanisms of *IGFBP2* in the adipogenic differentiation of MSCs. Our results show overexpression *IGFBP2* enhances adipogenic differentiation of WJCMSCs by activating JNK and Akt signaling pathway. Furthermore, we find that *IGFBP2* is negatively regulated by *BCOR*, which represses the adipogenic differentiation potential of WJCMSCs.

## Materials and methods

### Ethics statement and cell cultures

Between January and November 2012, patients were recruited from the Department of Oral and Maxillofacial Surgery of Beijing Stomatological Hospital, Capital Medical University. And human impacted third molars were collected from six healthy male patients (16–20 years old) under approved guidelines set by the Beijing Stomatological Hospital, Capital Medical University (Ethical Committee Agreement, Beijing Stomatological Hospital Ethics Review No. 2011–02), with written informed consent. In addition, we also obtained the informed consent from parent/guardian on behalf of minors (<18 years old). The authors had access to information that could identify individual participants during or after data collection.

Teeth were first disinfected with 75% ethanol and then washed with phosphate-buffered saline. PDLSCs were isolated, cultured, and identified as previously described [15,22]. Briefly, PDLSCs were separated from periodontal ligament in the middle one-third of the root. Subsequently, MSCs were digested in a solution of 3 mg/mL collagenase type I (Worthington Biochemical Corp., Lakewood, NJ, USA) and 4 mg/mL dispase (Roche Diagnostics Corp., Indianapolis, IN, USA) for 1 h at 37°C. Single-cell suspensions were obtained by cell passage through a 70-μm strainer (Falcon, BD Labware, Franklin Lakes, NJ, USA). Human BMSCs, ASCs, and WJCMSCs were purchased from ScienCell Research Laboratories (Carlsbad, CA, USA). MSCs were grown in a humidified, 5% CO_2_ incubator at 37°C in DMEM alpha modified Eagle’s medium (Invitrogen, Carlsbad, CA, USA), supplemented with 15% fetal bovine serum (FBS; Invitrogen, Carlsbad, CA, USA), 2 mmol/L glutamine, 100 U/mL penicillin and 100 μg/mL streptomycin (Invitrogen, Carlsbad, CA, USA). The culture medium was changed every 3 days. MSCs at passages 3–5 were used in subsequent experiments. Human embryonic kidney 293T cells were maintained in complete DMEM with 10% FBS, 100 U/mL penicillin, and 100 μg/mL streptomycin. For viral packaging, HEK293T cells at 80% confluency were co-transfected with plasmids and transfection reagent. For SP600125 (Cell Signaling Technology, Beverly, MA, USA), LY294002 (Cell Signaling Technology, Beverly, MA, USA), anisomycin (Cell Signaling Technology, Beverly, MA, USA), or insulin (Sigma-Aldrich, St. Louis, MO, USA) treatment, MSCs were starved for 24 h to synchronize the cells in DMEM alpha modified Eagle’s medium without serum, then changed to routine culture medium and treated with appropriate agents. The studies on human MSCs were conducted between May 2012 and November 2016. All cell-based experiments were repeated three times. http://dx.doi.org/10.17504/protocols.io.iegcbbw.

### Plasmid construction and viral infection

The plasmids were constructed using standard methods; all sequences were verified by appropriate restriction digestion and/or sequencing. Human full-length *IGFBP2* cDNA from ASCs fused to a M2-Flag tag was produced with a standard PCR protocol. This sequence (Flag-*IGFBP2*) was subcloned into the pQCXIN retroviral vector with AgeI and BglII restriction sites. Similarly, the human full-length *BCOR* cDNA was fused to a Flag tag (Flag-*BCOR*) and subcloned into the pQCXIN retroviral vector with AgeI and BamH1 restriction sites. For viral infections, MSCs were plated overnight, and then infected with retroviruses in the presence of polybrene (6 μg/mL, Sigma-Aldrich, St. Louis, MO, USA) for 12 h. After 48 h, infected cells were selected with 600 μg/mL G418 for 10 days. http://dx.doi.org/10.17504/protocols.io.iehcbb6.

### Western Blot analysis

Cells were lysed in RIPA buffer (10 mM Tris-HCl, 1 mM EDTA, 1% sodium dodecyl sulfate [SDS], 1% NP-40, 1:100 proteinase inhibitor cocktail, 50 mM β-glycerophosphate, 50 mM sodium fluoride). The samples were separated on a 10% SDS polyacrylamide gel and transferred to PVDF membranes with a semi-dry transfer apparatus (Bio-Rad, Hercules, CA, USA). The membranes were blotted with 5% dehydrated milk for 1 h and then incubated with primary antibodies overnight. The immune complexes were incubated with horseradish peroxidase-conjugated anti-rabbit or anti-mouse IgG (Promega, Madison, WI, USA) and visualized with SuperSignal reagents (Pierce, Rockford, IL, USA). Primary antibodies were purchased from following commercial sources: monoclonal anti-FLAG M2 (Clone No.9A3, Cat No.8146, Cell Signaling Technology, Beverly, MA, USA); monoclonal antibody against SAPK/JNK (Cat No. 9253, Cell Signaling Technology, Beverly, MA, USA); monoclonal antibody against phospho-SAPK/JNK (Cat No. 4668, Cell Signaling Technology, Beverly, MA, USA); monoclonal antibody against Akt (Cat No. 4685, Cell Signaling Technology, Beverly, MA, USA); polyclonal antibody against phospho-Akt (Cat No. 9271, Cell Signaling Technology, Beverly, MA, USA); monoclonal antibody against ERK1/2 and MAPK (Cat No. 4695, Cell Signaling Technology, Beverly, MA, USA); monoclonal antibody against phospho-p44/42 MAPK (Cat No. 4377, Cell Signaling Technology, Beverly, MA, USA); monoclonal antibody against p38 MAPK (Cat No. 8690, Cell Signaling Technology, Beverly, MA, USA); monoclonal antibody against phospho-p38 MAPK (Cat No. 4631, Cell Signaling Technology, Beverly, MA, USA). We also used a primary monoclonal antibody to detect the housekeeping protein, glyceraldehyde 3-phosphate dehydrogenase (GAPDH; Clone No. GAPDH 71.1, Cat No. G8795, Sigma-Aldrich, St. Louis, MO, USA). http://dx.doi.org/10.17504/protocols.io.iejcbcn.

### Oil Red O staining

Adipogenic differentiation was induced by using the StemPro adipogenesis differentiation kit (Invitrogen, Carlsbad, CA, USA). MSCs were grown in the adipose-inducing medium for 3 weeks. For Oil Red O staining, after induction, cells were fixed with 10% formalin for at least 1 h at room temperature. Next, cells were stained with the 60% Oil Red O in isopropanol as working solution for 10 min. The proportion of Oil Red O-positive cells was determined by counting stained cells under a light microscope. The final OD value in each group was normalized with the total protein concentrations prepared from a duplicate plate. http://dx.doi.org/10.17504/protocols.io.iemcbc6.

### Real-time RT-PCR

Total RNA was isolated from MSCs with TRIzol Reagent (Invitrogen, Carlsbad, CA, USA). Reverse transcription reactions contained 2 μg RNA, random hexamers or oligo (dT), and reverse transcriptase, and were performed according to the manufacturer’s protocol (Invitrogen, Carlsbad, CA, USA). Real-time PCR reactions were performed using the QuantiTect SYBR Green PCR kit (Qiagen, Hilden, Germany) and an Icycler iQ Multi-color Real-time PCR Detection System with the expression of *GAPDH* as the internal control. The primers used were: *IGFBP2*, forward, 5′-cgttcaagtgcaagatgtctctgaacg-3′ and reverse, 5′-ggatcagcttcccggtgttg-3′; *PPARγ*, forward, 5′-cgagaccaacagcttctccttctcg-3′ and reverse, 5′-tttcagaaatgccttgcagtgg-3′; *LPL*, forward, 5′-cggattaacattggagaagctatccg-3′ and reverse, 5′-agctggtccacatctccaagtc-3′; *CD36*, forward, 5′-cgattaacataagtaaagttgccataatcg-3′ and reverse, 5′-cgcagtgactttcccaataggac-3′; *CEBPA*, forward, 5′-cggcttatcctaaatactagagttgccg-3′ and reverse, 5′-ggacttggtgcgtctaagatga-3′; *GAPDH*, forward, 5’-cggaccaatacgaccaaatccg-3’ and reverse, 5’-agccacatcgctcagacacc-3’. The cycle threshold values (*Ct* values) were used to calculate the fold differences by the ΔΔ*Ct* method. http://dx.doi.org/10.17504/protocols.io.iencbde.

### Statistics

All statistical calculations were performed with SPSS20.0 statistical software (IBM, Armonk, NY). Comparisons between two groups were analysed by independent two-tailed Student’s *t*-tests, and comparisons between more than two groups were analysed by one-way ANOVA followed by a Duncan’s *post hoc* test. Data were expressed as the mean ± standard deviation (SD) of 3 experiments per group. *P* values < 0.05 were considered statistically significant.

## Results

### IGFBP2 promotes adipogenic differentiation of MSCs

**Fig 1:**
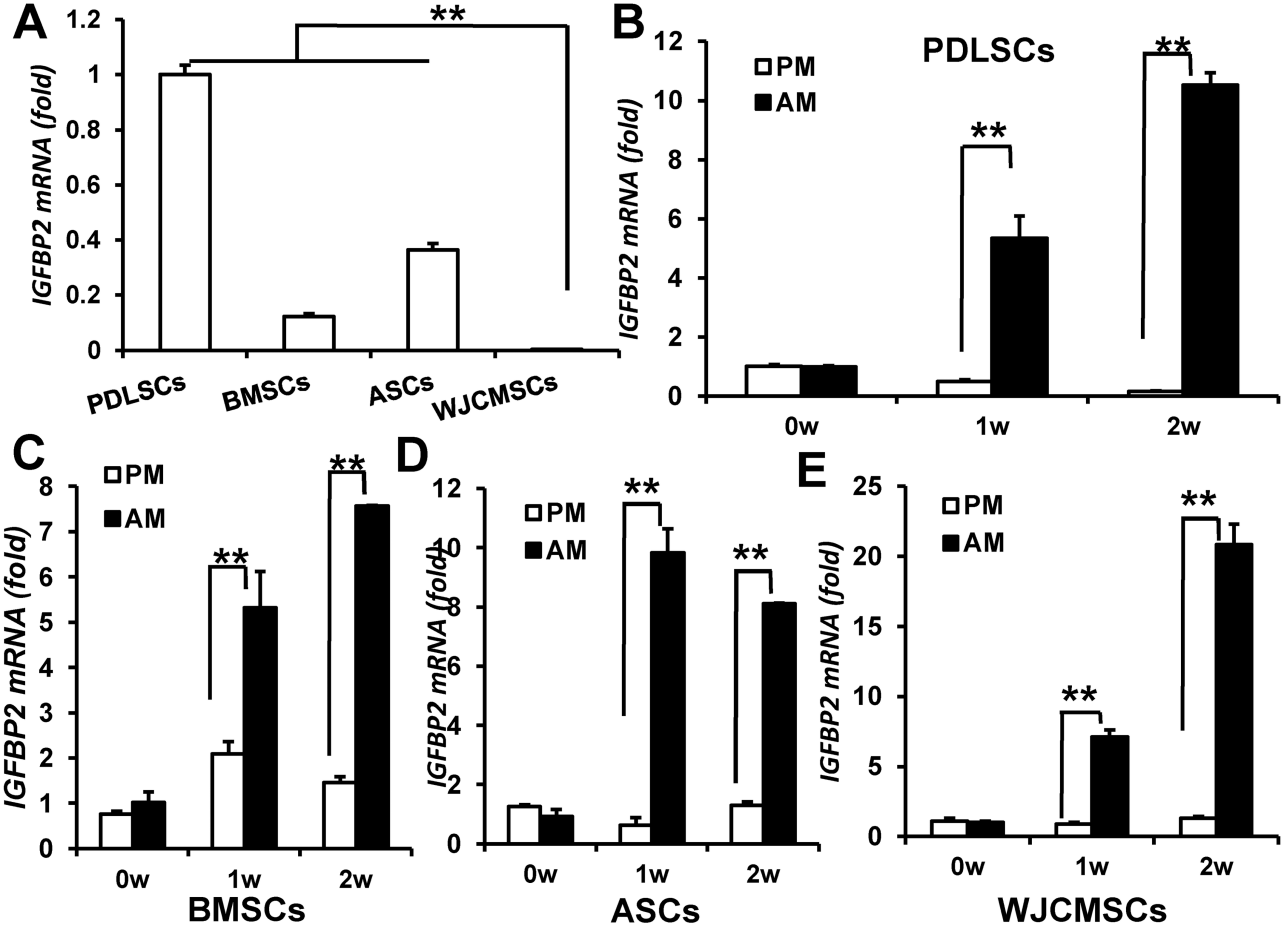
*IGFBP2* expression levels in MSCs. (A) Real-time RT-PCR revealed that lower *IGFBP2* expression in WJCMSCs than that in PDLSCs, BMSCs, and ASCs. (B-E) Increased *IGFBP2* expression after adipogenic induction in PDLSCs (B), BMSCs (C), ASCs (D), and WJCMSCs (E). *GAPDH* was used as an internal control. **p < 0.01. w: week; PM: proliferation medium; AM: adipose-inducing medium.

First, we used real-time RT-PCR to compare the *IGFBP2* mRNA levels in PDLSCs, BMSCs, ASCs, and WJCMSCs. We consistently found lower *IGFBP2* expression in WJCMSCs (0.00388±0.00033) than that in PDLSCs (1±0.0347), BMSCs (0.1225±0.011), and ASCs (0.364±0.023) (Fig 1A). Next, we investigated the *IGFBP2* expression upon adipogenic differentiation. Compared with proliferation medium, adipogenic-inducing medium induced upregulated *IGFBP2* expression in PDLSCs (Fig 1B), BMSCs (Fig 1C), ASCs (Fig 1D), and WJCMSCs (Fig 1E) at 1 and 2 weeks after induction.

**Fig 2:**
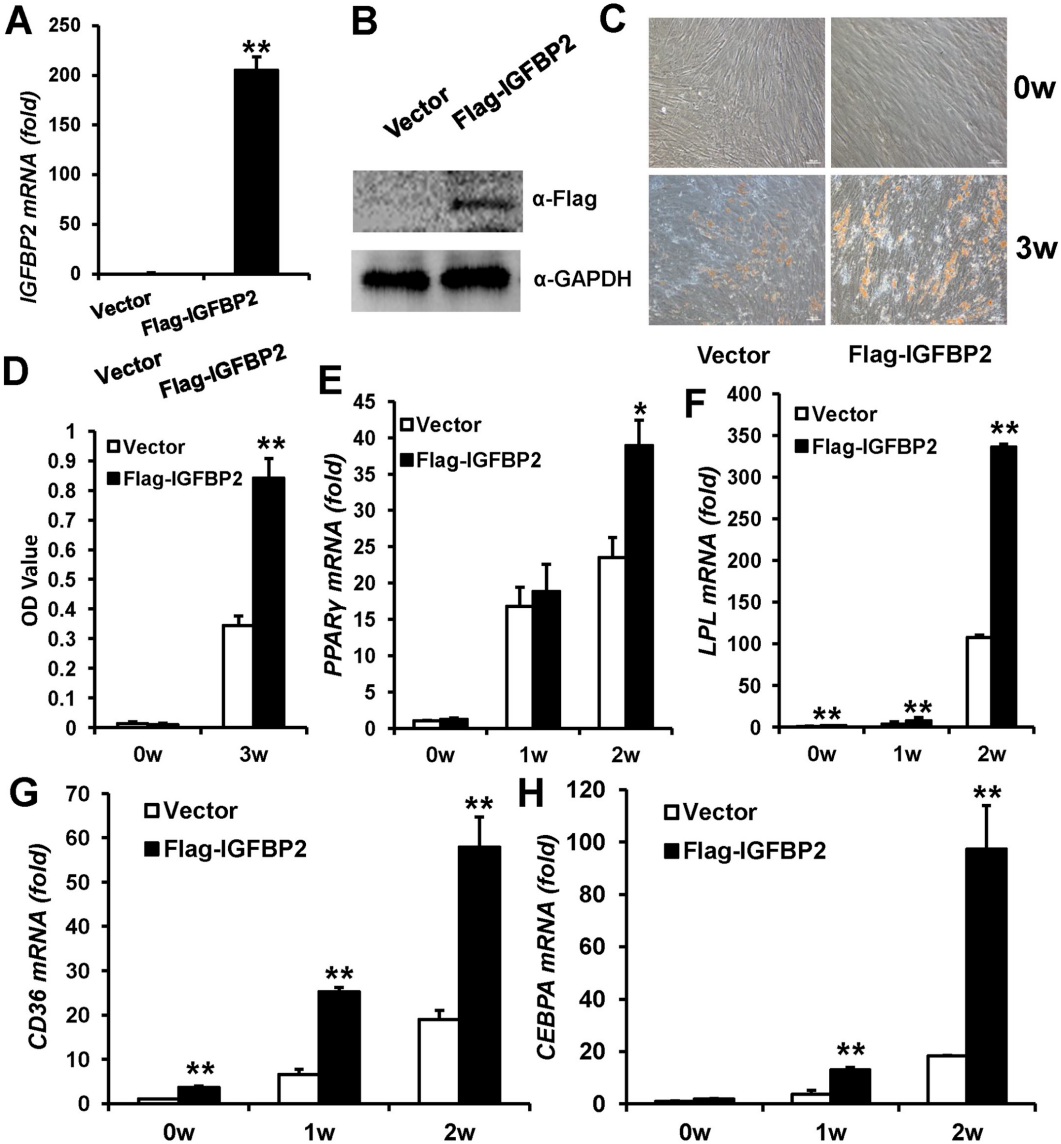
*IGFBP2* overexpression enhances adipogenic differentiation in WJCMSCs. (A) Flag-*IGFBP2*-infected WJCMSCs showed *IGFBP2* overexpression by real-time RT-PCR. *GAPDH* was used as an internal control. (B) Overexpression of IGFBP2 was verified by Western Blot analysis. (C-D) Oil Red O staining and quantitative analysis showed that *IGFBP2* overexpression prompted formation of lipid deposits. Scale bar: 100 μm. (E-H) Real-time RT-PCR showed that overexpression of *IGFBP2* upregulated expressions of *PPARγ* (E), *LPL* (F), *CD36* (G), and *CEBPA* (H) in WJCMSCs at 0, 1, and 2 weeks after induction. *GAPDH* was used as an internal control. *p < 0.05. **p < 0.01. α: anti; w: week.

To elucidate the function of *IGFBP2* in WJCMSCs, retrovirus expressing Flag-tagged wild type *IGFBP2* (Flag-*IGFBP2*) was used to perform a gain-of-function study in WJCMSCs. Ectopic IGFBP2 overexpression was verified by real-time RT-PCR (Fig 2A) and Western Blot (Fig 2B). To examine the adipogenic differentiation potential, transduced WJCMSCs were cultured in adipose-inducing medium. Following 3 weeks induction, Oil Red O staining showed significantly more lipid deposits in WJCMSC-Flag-*IGFBP2* cells than in WJCMSC-Vector cells (Fig 2C). After normalizing the data with total protein, these results suggested that WJCMSC-Flag-*IGFBP2* cells had stronger adipogenic differentiation potential (Fig 2D). We also examined the adipogenic differentiation markers: peroxisome proliferator-activated receptor γ (*PPARγ)*, lipoprotein lipase (*LPL*), CCAAT/enhancer-binding protein α (*CEBPA*), and cluster of differentiation 36 (*CD36*). The real-time PCR results indicated *IGFBP2*-overexpressing WJCMSCs (38.9±3.5) showed significantly higher *PPARγ* mRNA levels compared with cells infected with the empty vector (23.5±2.8) at 2 weeks following induction (Fig 2E). And the *LPL* (Fig 2F) and *CD36* (Fig 2G) mRNA levels were higher in WJCMSC-Flag-*IGFBP2* cells at 0, 1, and 2 weeks after induction compared with WJCMSC-Vector cells. At 2 and 3 weeks after induction, Flag-*IGFBP2*-overexpressing WJCMSCs also showed strong induction of the *CEBPA* (Fig 2H).

To determine whether IGFBP2 had similar functions in other MSCs, we overexpressed *IGFBP2* in BMSCs via retrovirus expressing Flag-tagged wild type *IGFBP2* (S1 Fig). Assessment of Oil Red O staining and real-time RT-PCR revealed that *IGFBP2* significantly promoted adipogenic differentiation in BMSCs (S1 Fig). Together, these results showed that *IGFBP2* overexpression substantially enhanced the adipogenic differentiation property of MSCs *in vitro*.

### IGFBP2 increases JNK and Akt phosphorylation in WJCMSCs

**Fig 3:**
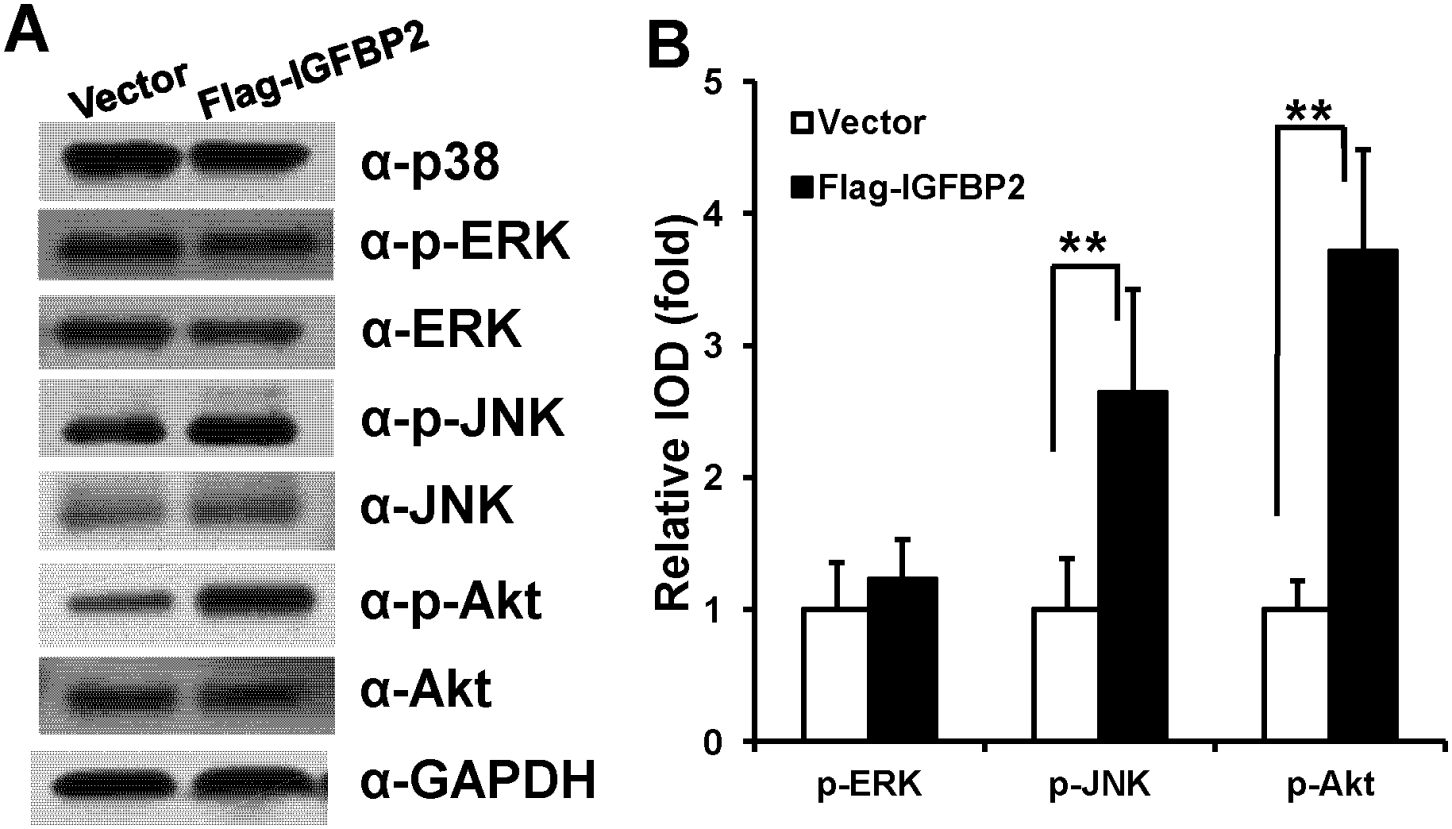
*IGFBP2* activates JNK and Akt signaling pathways. (A) Western Blot analysis demonstrated that *IGFBP2* overexpression caused an increase in p-JNK and p-Akt in WJCMSCs, however, the total amounts of JNK, ERK, p38, and Akt proteins were not affected; The phosphorylated p38 protein was not found. (B) Quantitative analysis of p-ERK, p-JNK, and p-Akt based on Western Blot results for the WJCMSC-Flag-*IGFBP2* cells and WJCMSC-Vector cells. Total ERK, JNK, and Akt were used as internal control respectively. **p < 0.01. α: anti.

To investigate how *IGFBP2* enhanced the adipogenic differentiation of WJCMSCs, we used Western Blot and quantitative analysis to examine the levels of proteins involved in MAPK signaling, including p38, ERK and JNK, and the Akt pathway. The results showed that overexpression of *IGFBP2* enhanced phosphorylation of JNK and phosphorylation of Akt in WJCMSCs, while phosphorylation of ERK, and total protein levels of p38, JNK, ERK, and Akt proteins were not affected (Fig 3A and 3B). And phosphorylated p38 MAPK was not found.

To test whether activated JNK or Akt had the capability of pro-adipogenesis in MSCs, we used the JNK activator (anisomycin) or Akt activator (insulin) in WJCMSCs. WJCMSCs were treated with 100nM, 200nM or 500nM anisomycin for 24 h to activate JNK signaling, or treated with 50nM, 100nM, 200nM or 500nM insulin for 24 h to activate Akt signaling. Western Blot results showed that 100nM, 200nM or 500nM anisomycin could effectively activate JNK signaling (S2 Fig), and 50nM, 100nM, 200nM or 500nM insulin could effectively activate Akt signaling (S2 Fig). Then, 100nM anisomycin and 50nM insulin were selected for further experiments. WJCMSCs were cultured in adipogenic-inducing medium with 100nM anisomycin or 50nM insulin. Three weeks after induction, Oil Red O staining and real-time RT-PCR results showed that 100nM anisomycin or 50nM insulin could significantly enhance the adipogenesis in WJCMSCs (S2 Fig).

### IGFBP2-enhanced adipogenic differentiation of WJCMSCs is repressed by JNK or Akt inhibitors

**Fig 4:**
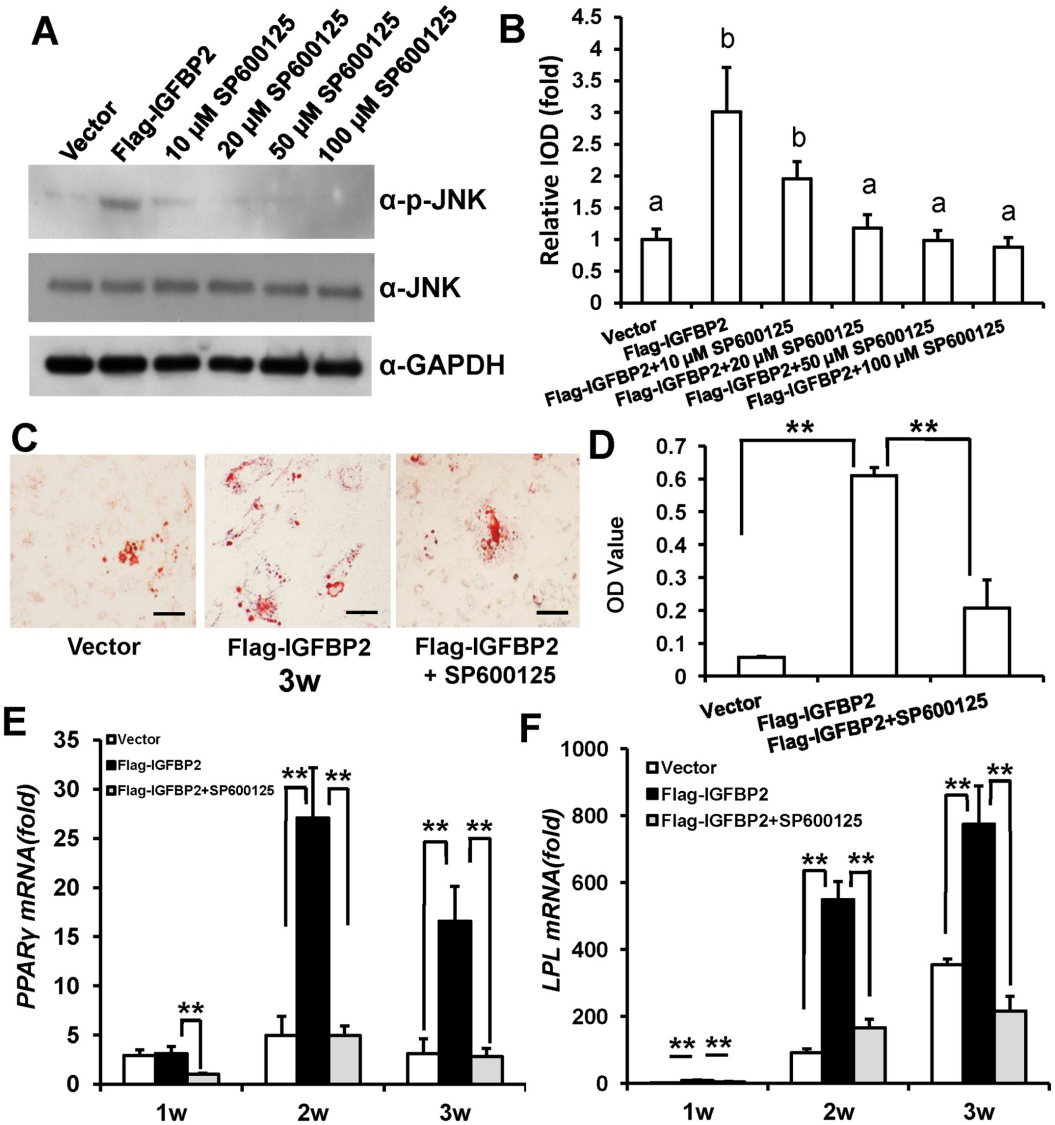
Effect of JNK inhibitor on *IGFBP2*-induced adipogenic differentiation of WJCMSCs. (A) Western Blot analysis showed a reduction of p-JNK in WJCMSC-Flag-*IGFBP2* after treatment with a JNK inhibitor, SP600125 (10 μM, 20 μM, 50 μM or 100 μM in DMSO) for 48 h during adipogenic induction. (B) Quantitative analysis of p-JNK based on Western Blot results. Total JNK was used as internal control. The expression levels that are indicated with the same letter do not differ significantly. (C-D) Oil Red O staining and quantitative analysis revealed that 20 μM SP600125 effectively suppressed *IGFBP2*-mediated enhancement of lipid formation. Scale bar: 100 μm. (E-F) Real-time RT-PCR results showed downregulated expressions of *PPARγ* (E) and *LPL* (F) in WJCMSC-Flag-*IGFBP2* cells following 20 μM SP600125 treatment during adipogenic induction at 1, 2, and 3 weeks. *GAPDH* was used as an internal control. **p < 0.01. α: anti; w: week.

First, WJCMSCs were treated with 10 μM, 20 μM, 50 μM or 100 μM specific JNK inhibitor, SP600125 for 48 h to block JNK signaling in WJCMSCs. Western Blot results (Fig 4A) and quantitative analysis (Fig 4B) indicated that 20 μM, 50 μM or 100 μM SP600125 could effectively block JNK signaling. Then, 20 μM SP600125 was selected for further experiments. Transduced WJCMSCs were cultured in adipogenic-inducing medium with 20 μM SP600125. Three weeks after induction, Oil Red O staining revealed that 20 μM SP600125 could restrain *IGFBP2*-mediated enhancement of adipogenic differentiation in WJCMSCs (Fig 4C). After normalizing the data with the total protein, the results indicated that the effect of *IGFBP2*-increased adipogenic differentiation of WJCMSCs was associated with JNK activation (Fig 4D). To confirm this finding, we further examined the adipogenic differentiation markers *PPARγ* and *LPL* by real-time RT-PCR. The results showed that *PPARγ* (Fig 4E) and *LPL* (Fig 4F) were significantly suppressed at 1, 2 or 3 weeks after induction in WJCMSC-Flag-*IGFBP2* + SP600125 group compared with WJCMSC-Flag-*IGFBP2* group.

**Fig 5:**
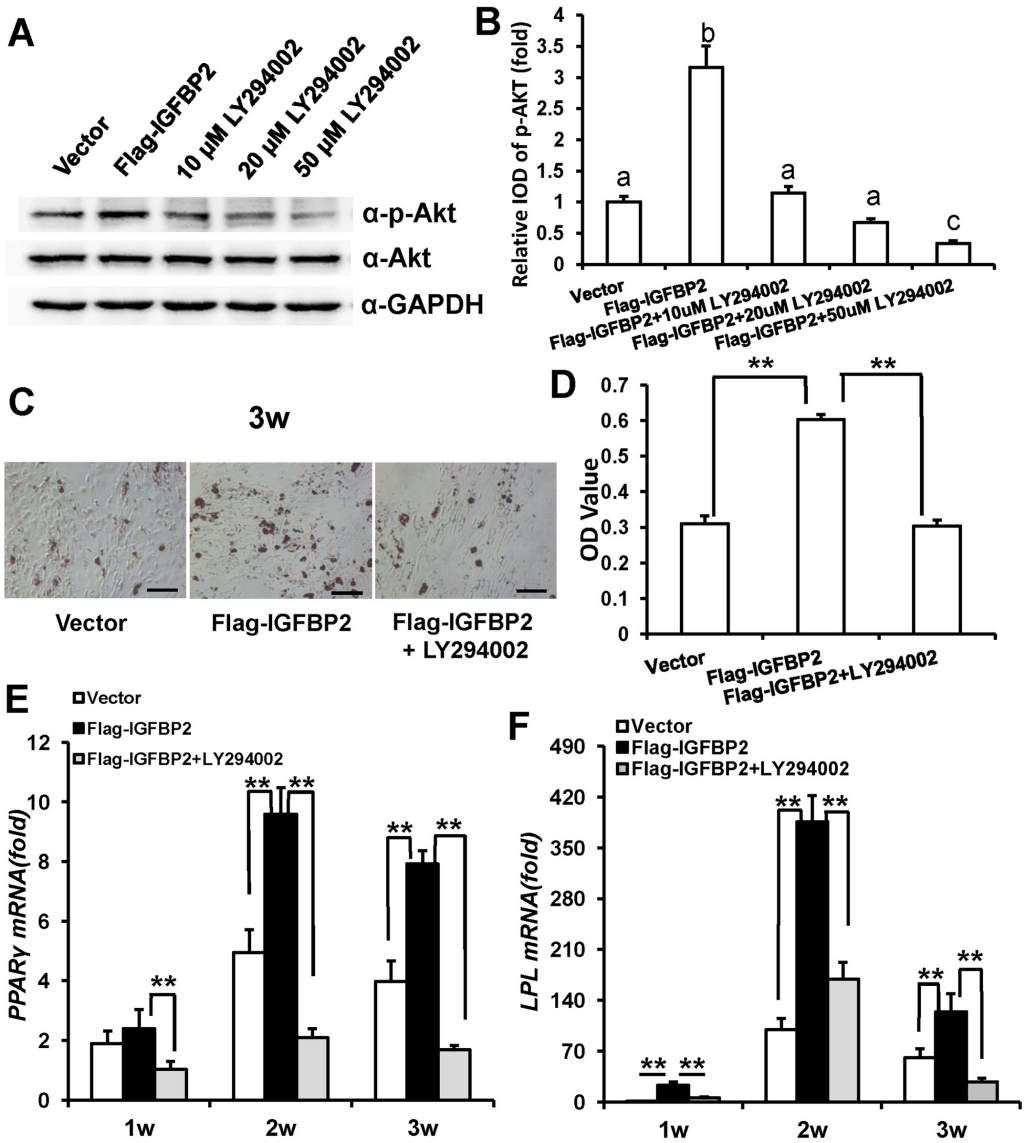
Effect of Akt inhibitor on *IGFBP2*-induced adipogenic differentiation of WJCMSCs. (A) Western Blotting results showed a reduction of p-Akt in WJCMSC-Flag-*IGFBP2* following treatment with an Akt inhibitor, LY294002 (10 μM, 20 μM, 50 μM or 80 μM in DMSO) for 1 h during adipogenic induction. (B) Quantitative analysis of p-Akt based on Western Blot results. Total Akt was used as internal control. The expression levels that are indicated with the same letter do not differ significantly. (C-D) Oil Red O staining and quantitative analysis showed that 10 μM LY294002 effectively inhibited *IGFBP2*-mediated adipogenic differentiation. Scale bar: 100 μm. (E-F) Real-time RT-PCR results showed downregulated expressions of *PPARγ* (E) and *LPL* (F) in WJCMSC-Flag-*IGFBP2* cells following 10 μM LY294002 treatment during adipogenic induction at 1, 2, and 3 weeks. *GAPDH* was used as an internal control. **p < 0.01. α: anti; w: week.

Then WJCMSCs were treated with specific Akt inhibitor, LY294002, to block Akt signaling for 1 h at concentration of 10 μM, 20 μM, 50 μM or 80 μM. Western Blot results (Fig 5A) and quantitative analysis (Fig 5B) suggested that 10 μM, 20 μM, 50 μM or 80 μM LY294002 could block Akt signaling efficiently. Then, 10 μM LY294002 was selected for further experiments. Compared with *IGFBP2*-infected cells, 10 μM LY294002 could inhibit *IGFBP2*-mediated enhancement of adipogenic differentiation in WJCMSCs by Oil Red O staining (Fig 5C) and quantitative lipid deposit measurements (Fig 5D). And real-time RT-PCR results showed that *PPARγ* (Fig 5E) and *LPL* (Fig 5F) were significantly suppressed at 1, 2 or 3 weeks after induction in LY294002 treated WJCMSC-Flag-*IGFBP2* group compared with untreated group.

### Activated Akt signaling by IGFBP2 is repressed by the specific JNK inhibitor in WJCMSCs

**Fig 6:**
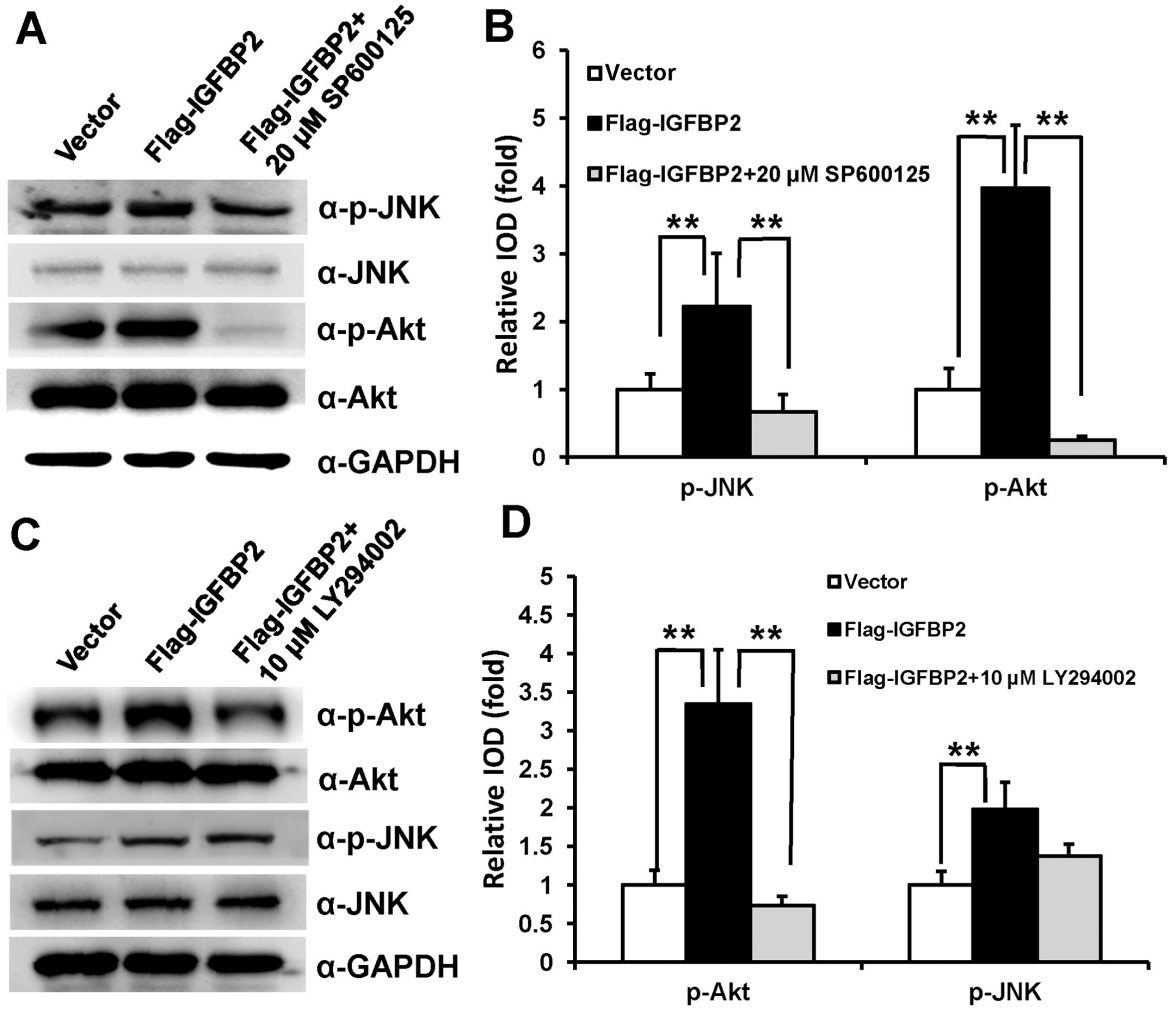
*IGFBP2*-mediated Akt activation is abrogated by JNK inhibitor. (A) Western Blot results indicated that administration of 20 μM SP600125 decreased JNK activation and abrogated Akt phosphorylation in WJCMSC-Flag-*IGFBP2* cells. (B) Quantitative analysis of p-JNK and p-Akt based on Western Blot results for the WJCMSC-Vector cells, WJCMSC-Flag-*IGFBP2* cells, and WJCMSC-Flag-*IGFBP2* + 20 μM SP600125 cells. Total Akt and JNK were used as internal control respectively. (C) Western Blotting results showed administration of 10 μM LY294002 decreased the level of p-Akt activation, while had no effect on JNK phosphorylation in WJCMSC-Flag-*IGFBP2* cells. (D) Quantitative analysis of p-Akt and p-JNK based on Western Blot results. Total Akt and JNK were used as internal control respectively. **p < 0.01. α: anti.

To further explore the underlying mechanism, we used the specific JNK inhibitor (20 μM SP600125) or Akt inhibitor (10 μM LY294002) to block the activated JNK or Akt pathway by *IGFBP2* in WJCMSCs. Western Blot results (Fig 6A) and quantitative analysis (Fig 6B) showed that 20 μM SP600125, which could inhibit the p-JNK level, effectively abrogated the expression of phosphorylation-Akt in WJCMSC-Flag-*IGFBP2* cells. However, treatment with 10 μM LY294002 had no significant effect on the expression of p-JNK in WJCMSC-Flag-*IGFBP2* cells (Fig 6C and 6D).

### BCOR negatively regulates IGFBP2 expression and inhibits adipogenic differentiation of WJCMSCs

**Fig 7:**
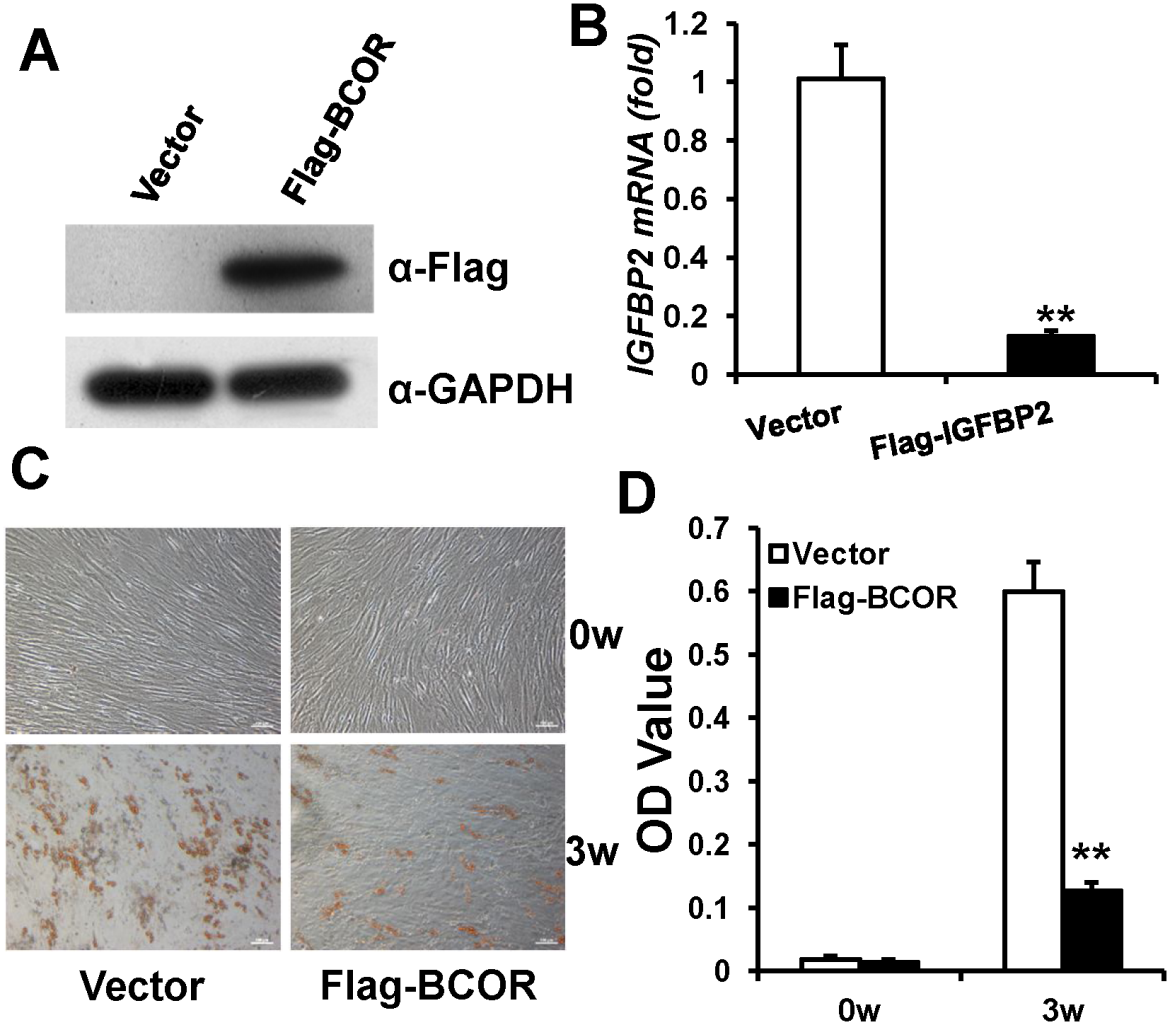
*BCOR* decreases *IGFBP2* expression and weakens adipogenic differentiation in WJCMSCs. (A) Flag-*BCOR*-infected WJCMSCs showed BCOR overexpression, as determined by Western Blot analysis. GAPDH was used as an internal control. (B) Real-time RT-PCR analysis showed that *BCOR* overexpression suppressed the expression of *IGFBP2* in WJCMSCs. *GAPDH* was used as an internal control. (C-D) Oil Red O staining and quantitative analysis showed that *BCOR* overexpression inhibited the formation of lipid deposits. Scale bar: 500 μm (a, b), 100μm (c, d). **p < 0.01. α: anti; w: week.

Ectopic BCOR overexpression was confirmed by Western Blot analysis (Fig 7A). Real-time RT-PCR results showed that *BCOR* overexpression in WJCMSCs suppressed the expression of *IGFBP2* (Fig 7B). Next, to investigate adipogenic differentiation, WJCMSCs were cultured in adipogenic-inducing medium. After induction for 3 weeks, Oil Red O staining (Fig 7C) and quantitative lipid deposit measurements (Fig 7D) showed there were significantly fewer lipid deposits in WJCMSC-Flag-*BCOR* cells than in WJCMSC-Vector cells.

## Discussion

Mesenchymal stem cells derived from Wharton’s jelly of the umbilical cord, which are usually discarded after birth, possess multipotent abilities between those of embryonic and adult stem cells [2,45]. Studies have shown that WJCMSCs possess many attractive properties, including expanding faster than adult-derived MSCs, ample cell supply and the potential for autologous grafting if they are cryopreserved for future use [7,10,12]. Moreover, in clinical practice, WJCMSCs have been successfully used to treat autoimmune disease [11]. Our previous study found that compared with PDLSCs, WJCMSCs exhibited decreased adipogenic differentiation potential, and unmodified WJCMSCs might not be good seed cells for tissue regeneration [15]. Therefore, a critical issue for WJCMSCs applied in tissue engineering is how to enhance the differentiation potentials and regenerative abilities. Using microarray analysis, we observed decreased expression of *IGFBP2* in WJCMSCs compared with PDLSCs [15]. IGFBP2 is mainly expressed in highly proliferative fetal tissues which represent extensive cell movement and tissue remodeling [46]. Our results confirm that *IGFBP2* is a potential mediator for enhancing adipogenic differentiation of WJCMSCs and BMSCs. The potentiality of *IGFBP2* and the possibility of modulating specific pathways underlying biological process of WJCMSCs offer new strategies in the field of regenerative medicine.

Regulation of adipogenic differentiation by growth factors is a complex process [20,47,48]. *PPARγ* is a master regulator of adipogenesis, and generally most all pro-adipogenic signaling pathways associated with *PPARγ* [49]. JNK is one of the major sub families of MAPKs [25–27]. Studies revealed that JNK pathway was associated with regulating adipogenic differentiation. Previous research showed that wild-type *IGFBP2*-overexpressing cells showed a higher level of phosphorylation JNK [42]. In NIH-OVCAR3 cells, *IGFBP2* promoted proliferation, potentiated ERK phosphorylation and activated SAPK/JNK signaling pathway [43]. Moreover, the anti-adipogenesis effect of 6-thioinosine was mediated by decreased expression of *PPARγ* through JNK pathway. Loss of JNK1 activity resulted in resistance to high-fat diet-induced obesity *in vivo* [50,51]. In addition, Akt was also essential for inducing *PPARγ* and adipogenic differentiation; depletion of *Akt* impaired adipogenesis in mice [39–41]. Furthermore, impaired IGF1 mitogenesis involving the Akt pathway contributed to the distinct growth phenotype of visceral preadipocytes. More importantly, many researches inferred that using the JNK specific inhibitor or siRNA led to decreased Akt phosphorylation in many cells and cell processes [52–54]. However, it was reported that activation of JNK decreased Akt phosphorylation in liver tissue [42,43]. Pretreatment with the JNK specific inhibitor and salvianolic acid A caused decreased p-JNK and increased p-Akt in diabetic rats with ischemia/reperfusion [55]. Our results show that *IGFBP2* overexpression activates phosphorylation of JNK and Akt signaling, and activated JNK or Akt signaling enhances adipogenic differentiation of MSCs. In addition, JNK or Akt inhibitor suppresses *IGFBP2*-mediated enhancement of adipogenic differentiation in WJCMSCs. Separately, the results indicated that JNK and Akt signaling pathway exert an important role for *IGFBP2*-enhanced adipogenic differentiation. Furthermore, the specific JNK inhibitor markedly decreases the expression of phosphorylated Akt activated by *IGFBP2*, indicating that Akt is the downstream of the JNK in *IGFBP2* mediated signaling cascade. Taken together, our results confirm that *IGFBP2* enhances adipogenic differentiation of WJCMSCs via activated JNK/Akt signaling pathway. However, further study is required to investigate the regulation mechanism about JNK/Akt crosstalk in the process.

In addition to these results, we also find that *BCOR* negatively regulates the expression of *IGFBP2*; this is consistent with our previous microarray analysis, which found that *IGFBP2* was highly expressed in stem cells from the apical papilla (SCAPs) from oculo-facio-cardio-dental (OFCD) syndrome that had a mutation in *BCOR* [56]. Our results also reveal that *BCOR* represses adipogenic differentiation of WJCMSCs. The *BCOR* gene encodes a protein known as the *BCL6* co-repressor, which might use an epigenetic mechanism to direct gene silencing [56–58]. Previous researches inferred that *BCOR* regulated the function of MSCs by associating with the activating enhancer binding protein 2 alpha (*AP2*α) promoter [56]. The 5' flanking region of *IGFBP2* gene contains motifs that might be recognized by transcription factor *AP2* [59]. Based on those studies, we speculate *BCOR* may be involved in the regulation of *IGFBP2* by epigenetics or *AP2*. However, this is beyond the scope of the current study and will require further investigation.

In summary, our results identify a novel function of *IGFBP2* in adipogenic differentiation of MSCs. Generally, *BCOR* negatively regulates *IGFBP2*, and overexpression of *IGFBP2* can enhance the adipogenic differentiation of WJCMSCs through activating JNK and Akt signaling pathways. This study elucidates molecular mechanisms underlying adipogenic differentiation of WJCMSCs, and suggests that *IGFBP2* may be a potential target to promote the adipose tissue regeneration.

## Supporting information

S1 Fig *IGFBP2* overexpression enhances adipogenic differentiation in BMSCs. (A) Flag-*IGFBP2*-infected BMSCs showed *IGFBP2* overexpression by Real-time RT-PCR. (B-C) Oil Red O staining and quantitative analysis showed that *IGFBP2* overexpression prompted formation of lipid deposits. Scale bar: 100 μm. (D-E) Real-time RT-PCR showed that overexpression of *IGFBP2* upregulated expressions of *PPARγ* (D) and *LPL* (E) in BMSCs after induction. *GAPDH* was used as an internal control. **p < 0.01. α: anti; w: week.
(TIF)

S2 Fig (A) Western Blotting results showed an accumulation of p-JNK in WJCMSCs following treatment with the JNK activator, anisomycin (100 nM, 200 nM or 500 nM in ethanol) for 24 h during adipogenic induction. (B) Quantitative analysis of p-JNK based on Western Blot results. Total JNK was used as internal control. The expression levels that are indicated with the same letter do not differ significantly. (C) Western Blotting results showed an accumulation of p-Akt in WJCMSCs following treatment with the Akt activator, insulin (50 nM, 100 nM, 200 nM or 500 nM in culture medium) for 24 h during adipogenic induction. (D) Quantitative analysis of p-Akt based on Western Blot results. Total Akt was used as internal control. The expression levels that are indicated with the same letter do not differ significantly. (E-F) Oil Red O staining and quantitative analysis showed that 100 nM anisomycin or 50 nM insulin prompted formation of lipid deposits. Scale bar: 100 μm. (G-H) Real-time RT-PCR results showed upregulated expressions of *PPARγ* (G) and *LPL* (H) in WJCMSC cells following 100 nM anisomycin or 50 nM insulin treatment during adipogenic induction at 0 and 3 weeks. *GAPDH* was used as an internal control. **p < 0.01. α: anti; w: week.
(TIF)

S1 Data This file contains all the primary data of the results in this manuscript. (ZIP)

## Abbreviations

Akt: protein kinase B
AM: adipose-inducing medium
AP2α: activating enhancer binding protein 2 alpha
ASCs: adipose-derived stem cells
BCOR: BCL6 corepressor
BMSCs: bone marrow mesenchymal stem cells
CD36: 36
CEBPA: CCAAT/enhancer-binding protein α
CREB: cAMP response element-binding protein
ERK: extracellular regulated protein kinases
GAPDH: glyceraldehyde-3-phosphate dehydrogenase
IGF: insulin-like growth factor
IGFBP: insulin-like growth factor binding protein
JNK: c-Jun N-terminal kinase
LPL: lipoprotein lipase
MAPK: mitogen-activated protein kinase
MSCs: mesenchymal stem cells
OFCD: oculo-facio-cardio-dental
PDLSCs: periodontal ligament stem cells
PM: proliferation medium
PPARγ: peroxisome proliferator-activated receptor γ
SCAPs: stem cells from apical papilla
WJCMSCs: Wharton’s jelly of the umbilical cord mesenchymal stem cells
