# Supplementary figures and images for: IGFBP2 enhances adipogenic differentiation potentials of mesenchymal stem cells from Wharton's jelly of the umbilical cord via JNK and Akt signaling pathways
Source: PLoS One. 2017 Aug 31;12(8):e0184182. doi: 10.1371/journal.pone.0184182 (PMC5578624; doi:10.1371/journal.pone.0184182)

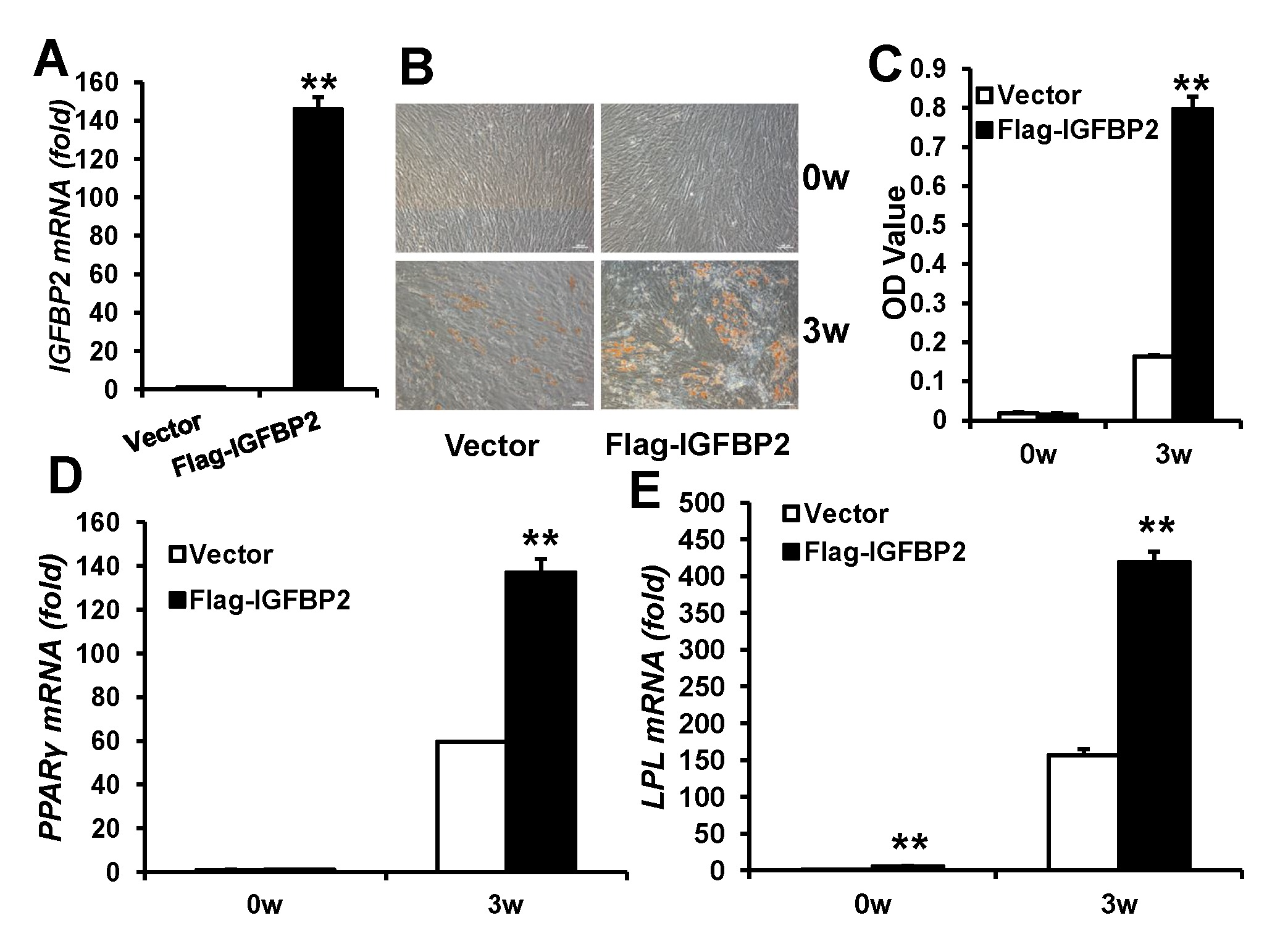

Supplement: S1 Fig — (A) Flag-IGFBP2-infected BMSCs showed IGFBP2 overexpression by Real-time RT-PCR. (B-C) Oil Red O staining and quantitative analysis showed that IGFBP2 overexpression prompted formation of lipid deposits. Scale bar: 100 μm. (D-E) Real-time RT-PCR showed that overexpression of IGFBP2 upregulated expressions of PPARγ (D) and LPL (E) in BMSCs after induction. GAPDH was used as an internal control. **p < 0.01. α: anti; w: week. (TIF) [file pone.0184182.s001.tif]

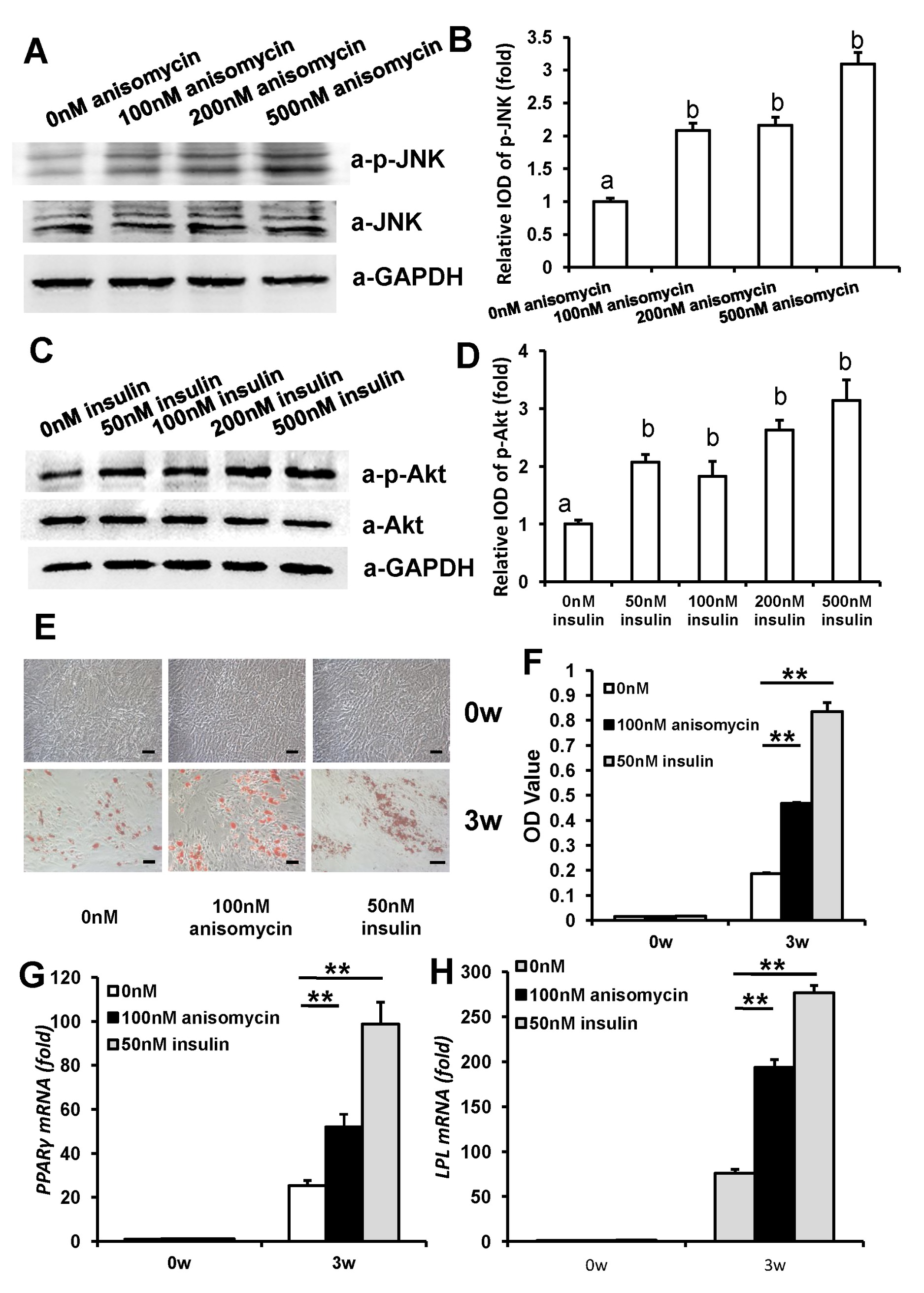

Supplement: S2 Fig — (A) Western Blotting results showed an accumulation of p-JNK in WJCMSCs following treatment with the JNK activator, anisomycin (100 nM, 200 nM or 500 nM in ethanol) for 24 h during adipogenic induction. (B) Quantitative analysis of p-JNK based on Western Blot results. Total JNK was used as internal control. The expression levels that are indicated with the same letter do not differ significantly. (C) Western Blotting results showed an accumulation of p-Akt in WJCMSCs following treatment with the Akt activator, insulin (50 nM, 100 nM, 200 nM or 500 nM in culture medium) for 24 h during adipogenic induction. (D) Quantitative analysis of p-Akt based on Western Blot results. Total Akt was used as internal control. The expression levels that are indicated with the same letter do not differ significantly. (E-F) Oil Red O staining and quantitative analysis showed that 100 nM anisomycin or 50 nM insulin prompted formation of lipid deposits. Scale bar: 100 μm. (G-H) Real-time RT-PCR results showed upregulated expressions of PPARγ (G) and LPL (H) in WJCMSC cells following 100 nM anisomycin or 50 nM insulin treatment during adipogenic induction at 0 and 3 weeks. GAPDH was used as an internal control. **p < 0.01. α: anti; w: week. (TIF) [file pone.0184182.s002.tif]
