# Supplementary material for: IGFBP2 enhances adipogenic differentiation potentials of mesenchymal stem cells from Wharton's jelly of the umbilical cord via JNK and Akt signaling pathways
Source: PLoS One. 2017 Aug 31;12(8):e0184182. doi: 10.1371/journal.pone.0184182 (PMC5578624; doi:10.1371/journal.pone.0184182)
Supplement: S1 Data — (ZIP) [file pone.0184182.s003.zip › primary data/western blot data .pdf]

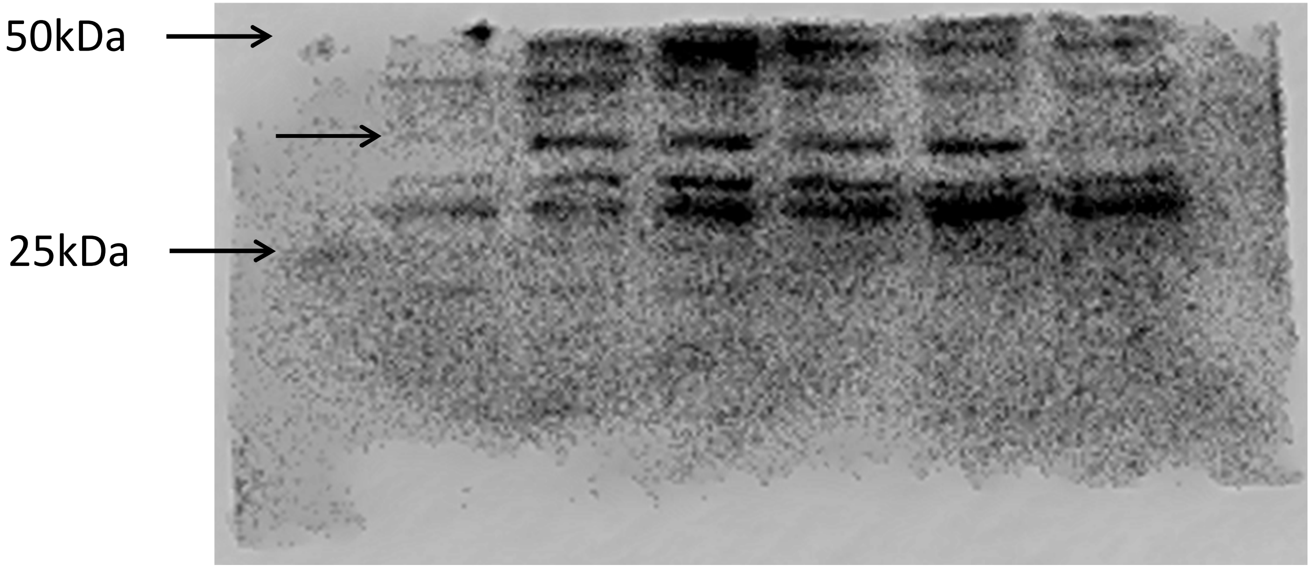

Fig 2B Flag

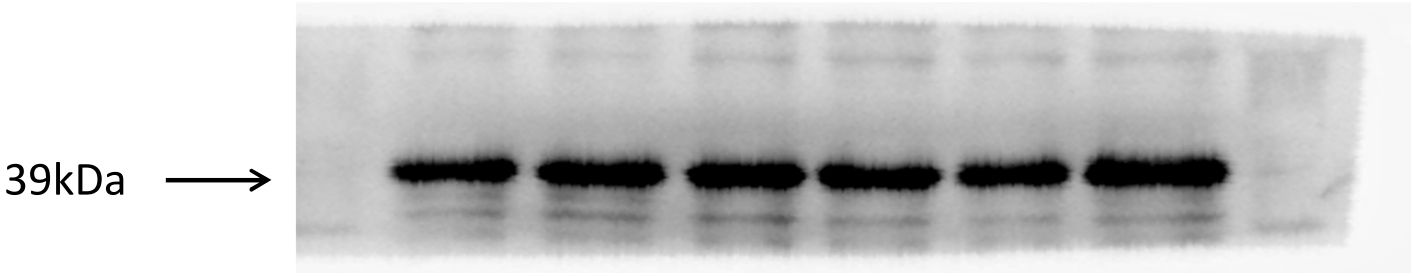

Fig 2B GAPDH

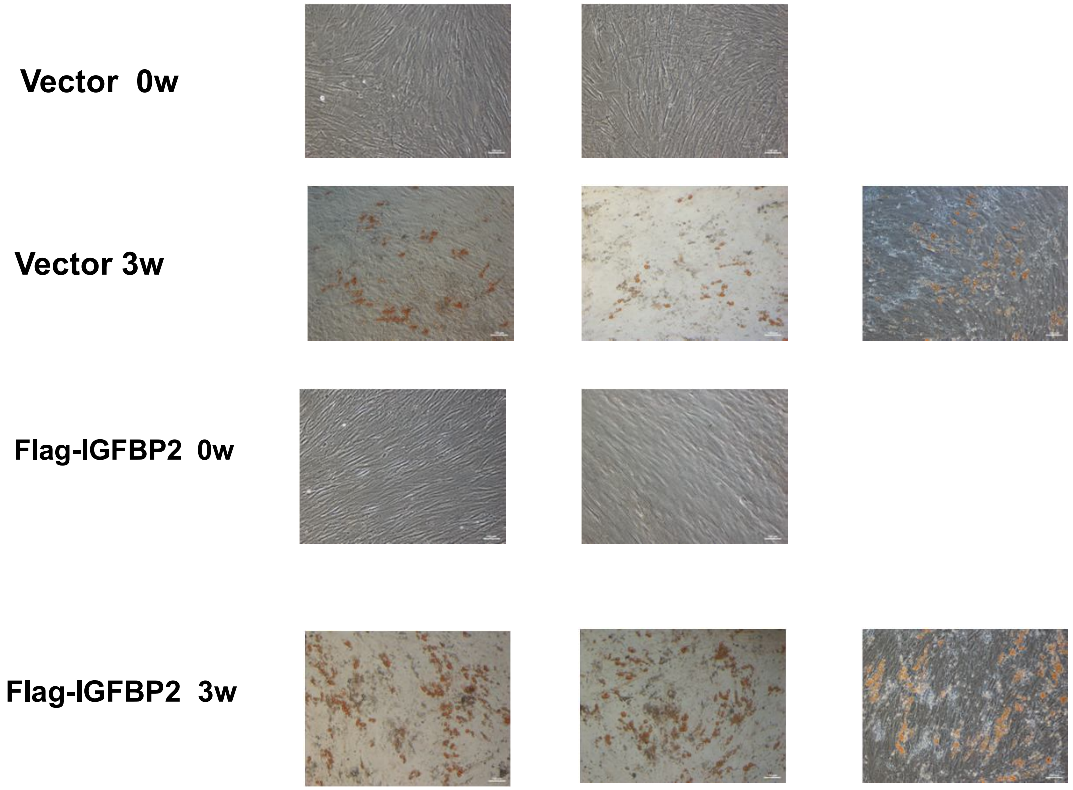

Fig 2C

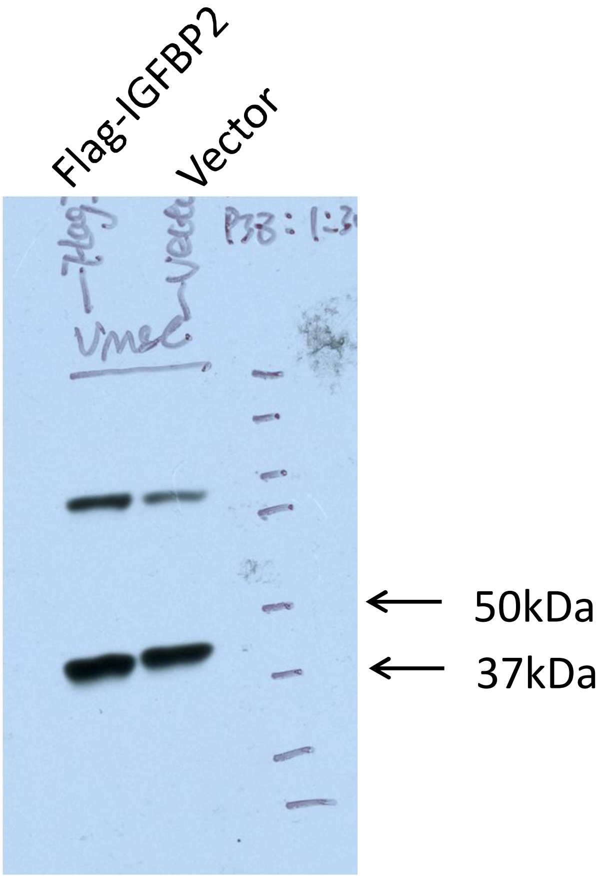

Fig 3A p38

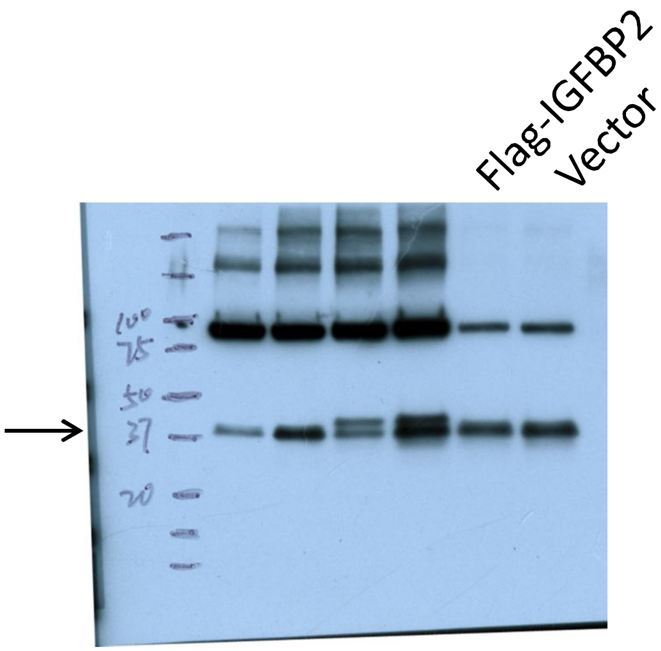

Fig 3A p-ERK

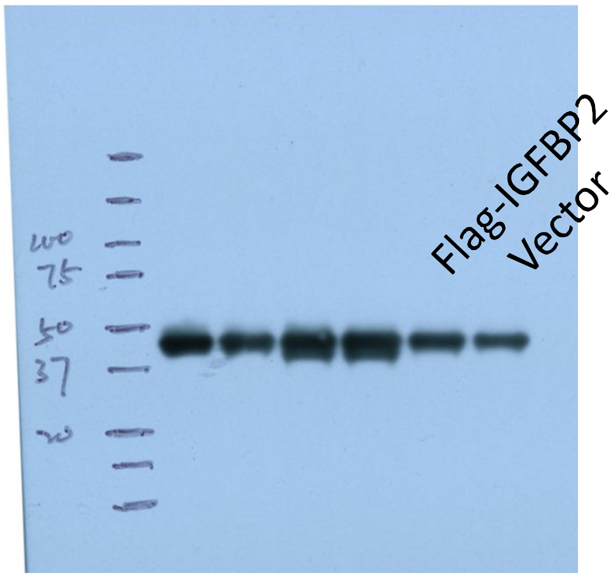

Fig 3A ERK

Other Lanes: IGFBPs modified MSCs

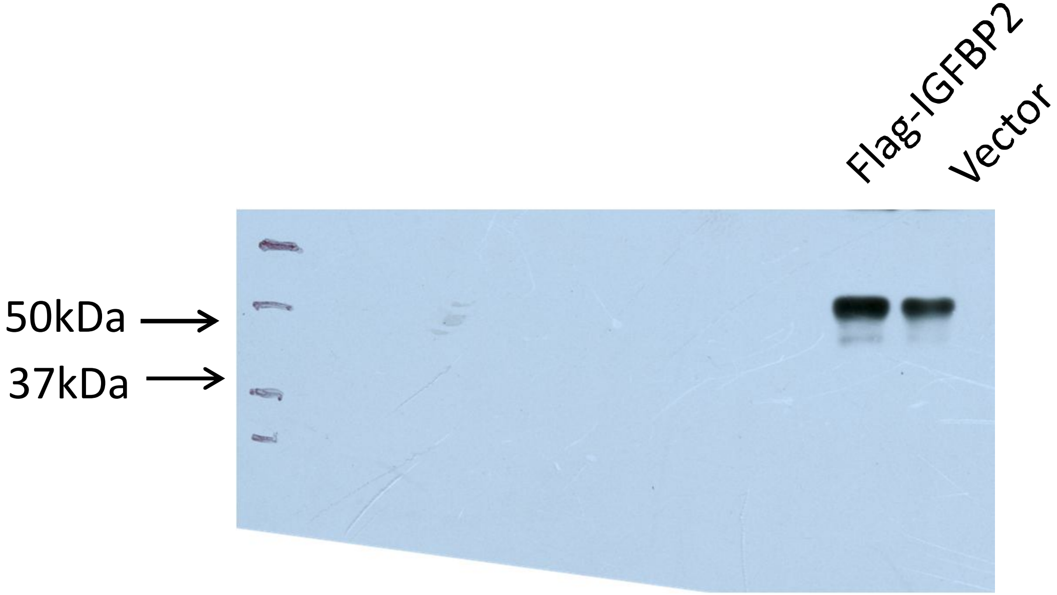

Other Lanes: IGFBPs modified MSCs

Fig 3A p-JNK

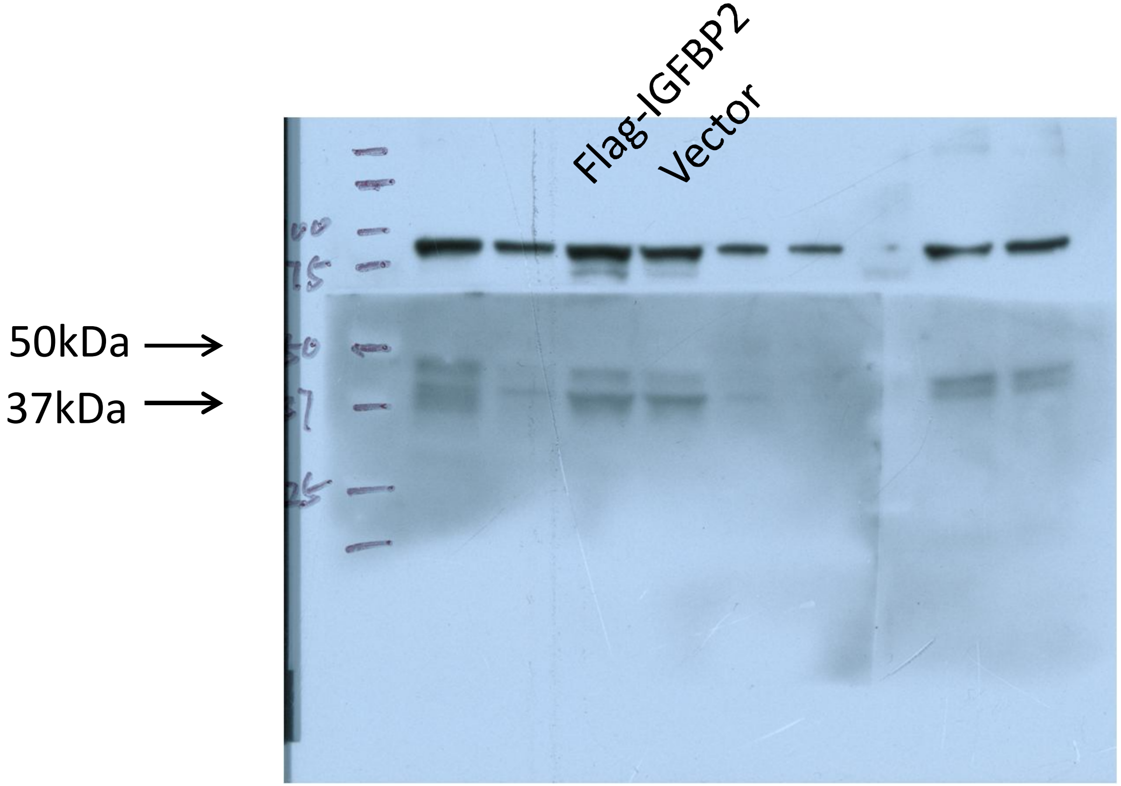

Other Lanes: IGFBPs modified MSCs

Fig 3A JNK

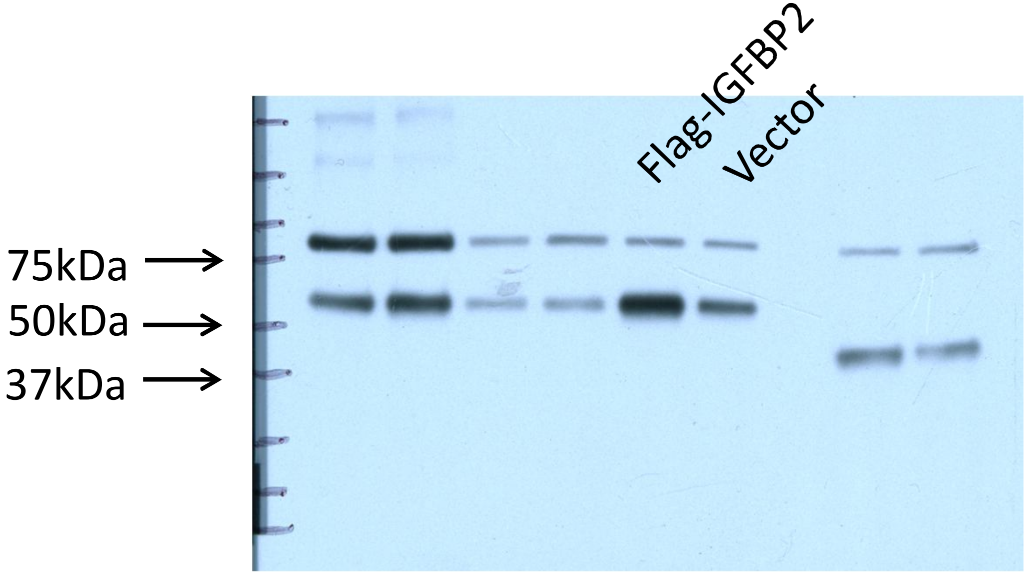

Other Lanes: IGFBPs modified MSCs

Fig 3A p-Akt

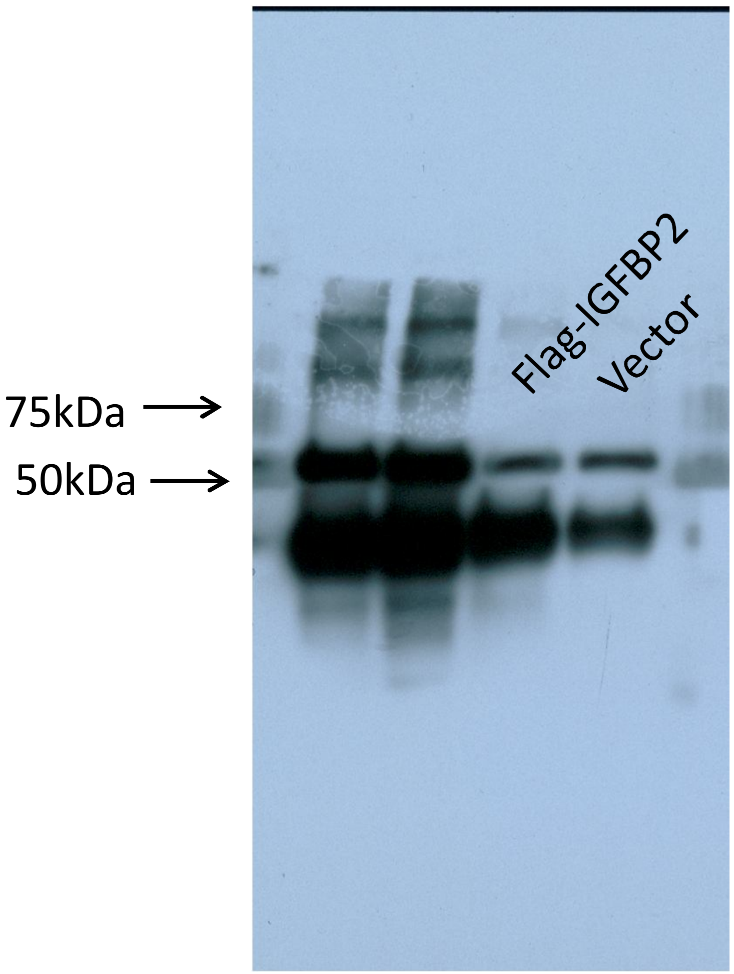

Other Lanes: IGFBPs modified MSCs

Fig 3A Akt

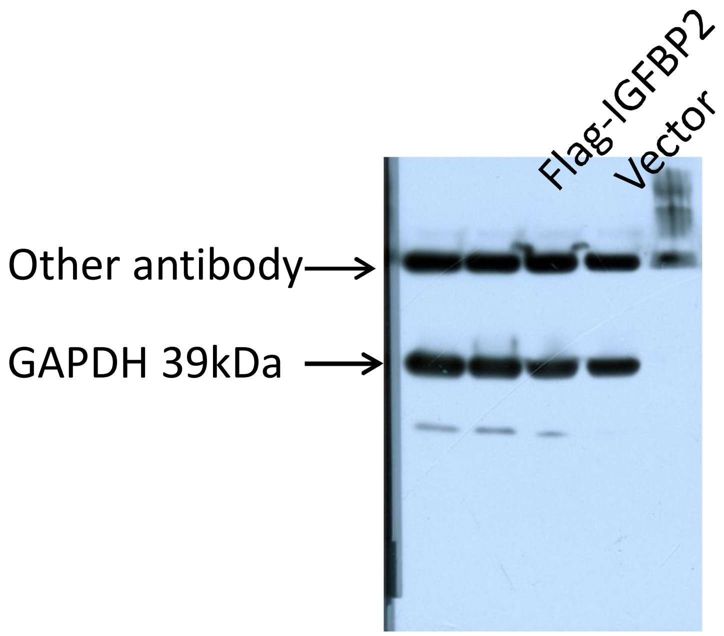

Other Lanes: IGFBPs modified MSCs

Fig 3A GAPDH

p-JNK →

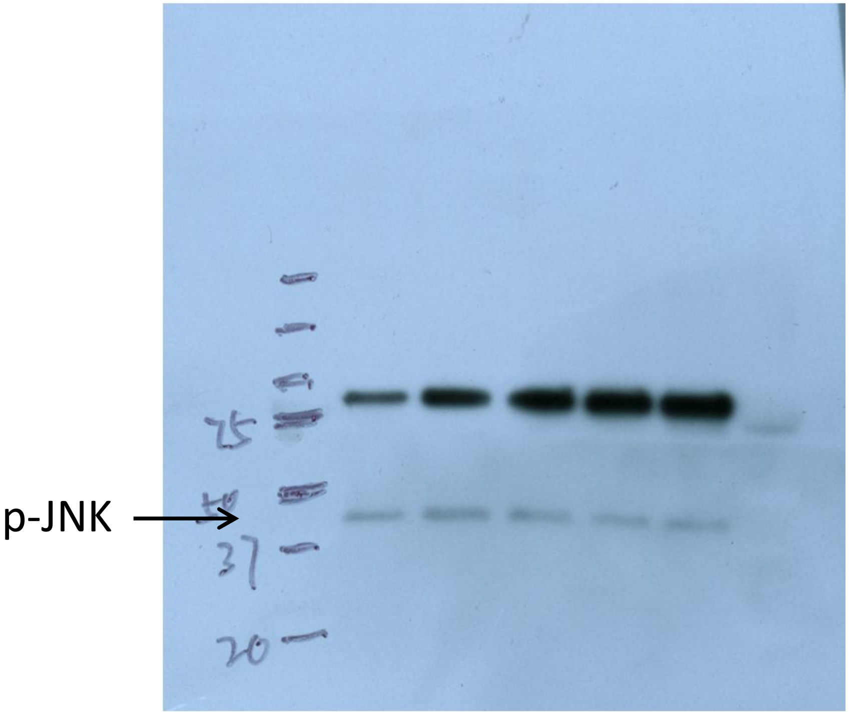

Fig 4A p-JNK

JNK

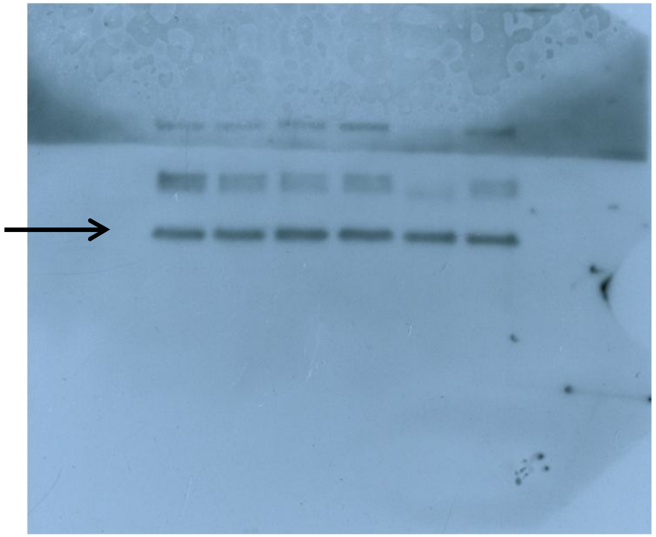

Fig 4A JNK

GAPDH →

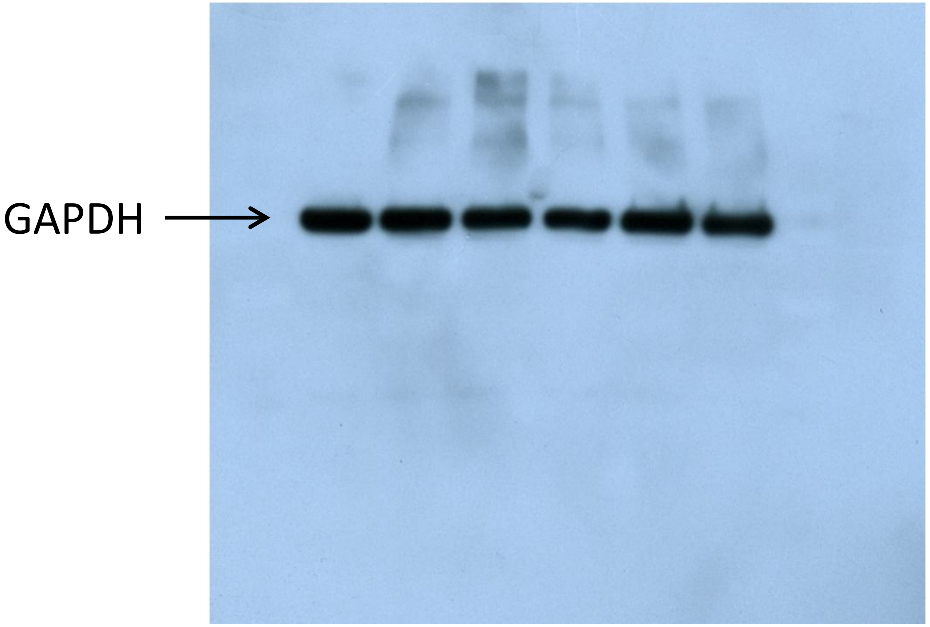

Fig 4A GAPDH

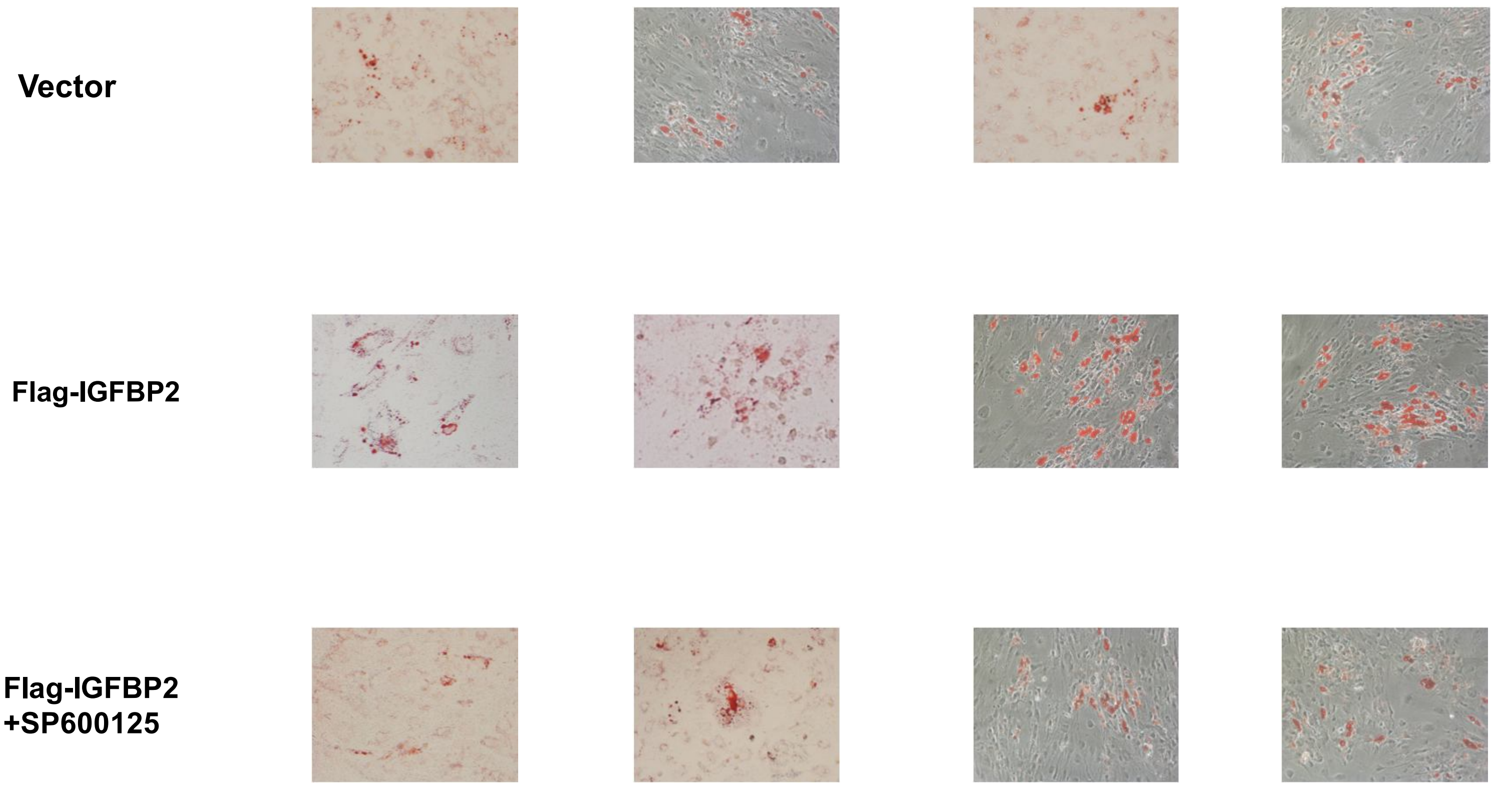

Fig 4A C

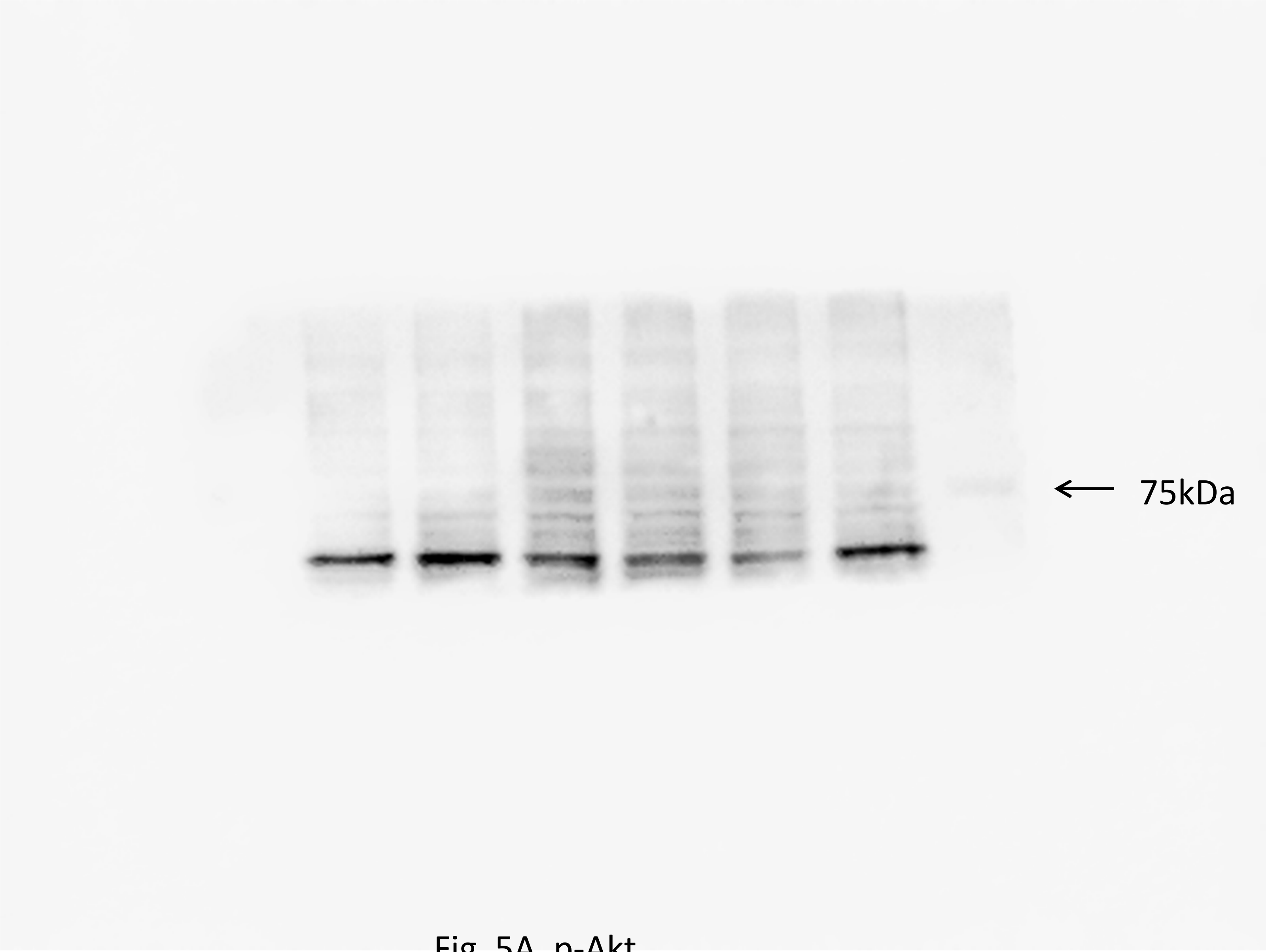

Fig 5A p-Akt

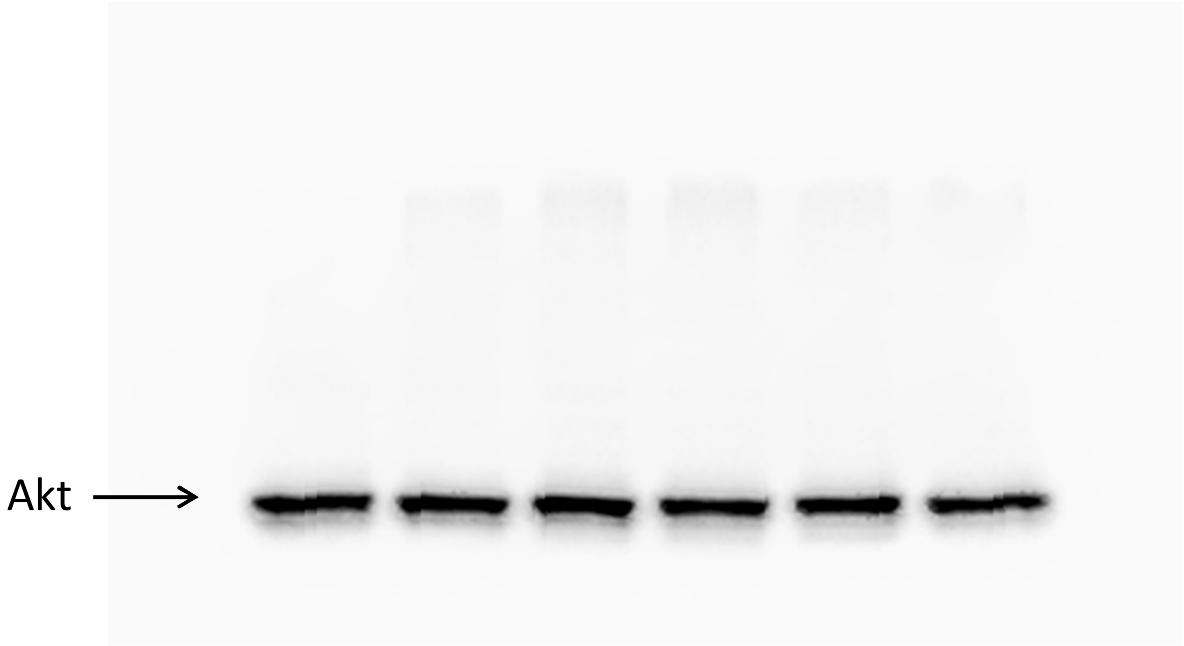

Fig 5A Akt

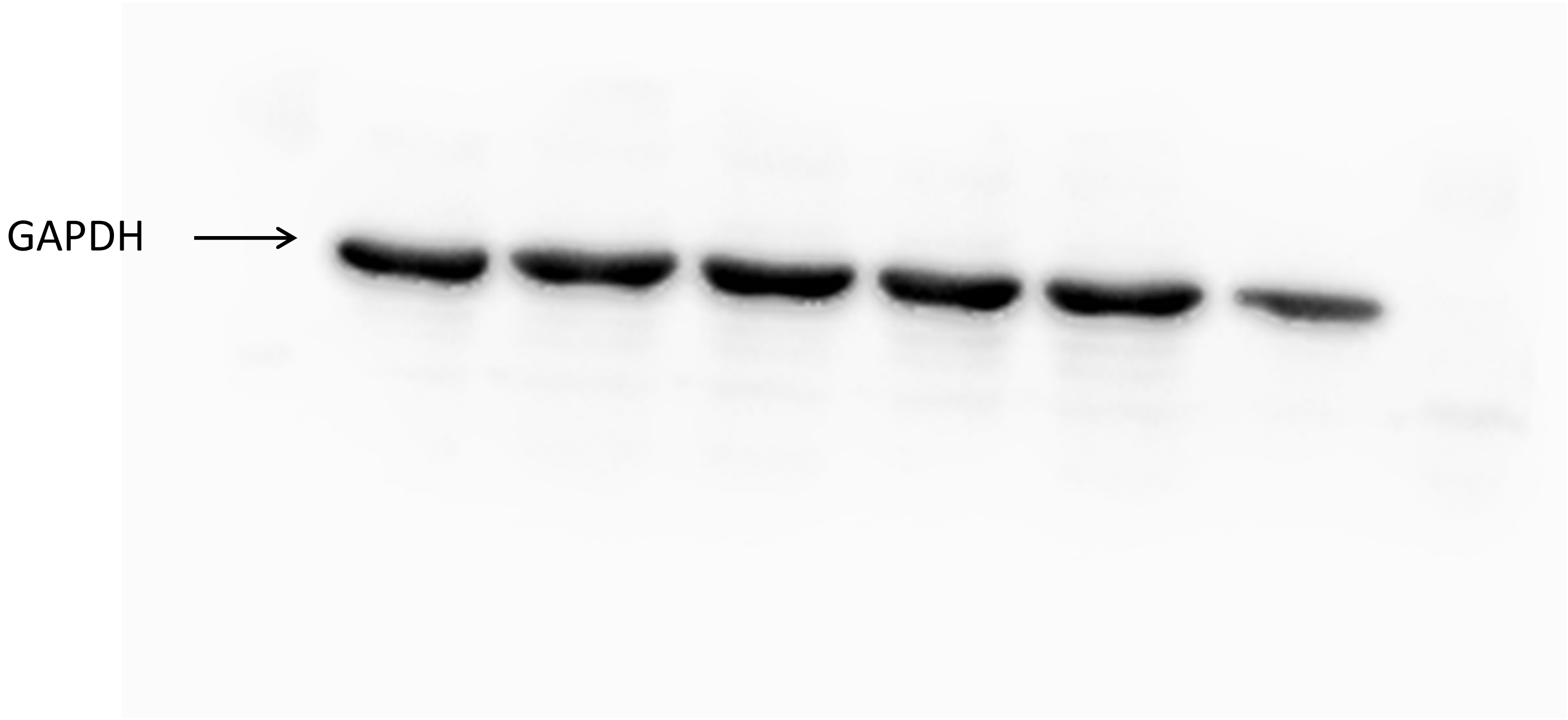

Fig 5A GAPDH

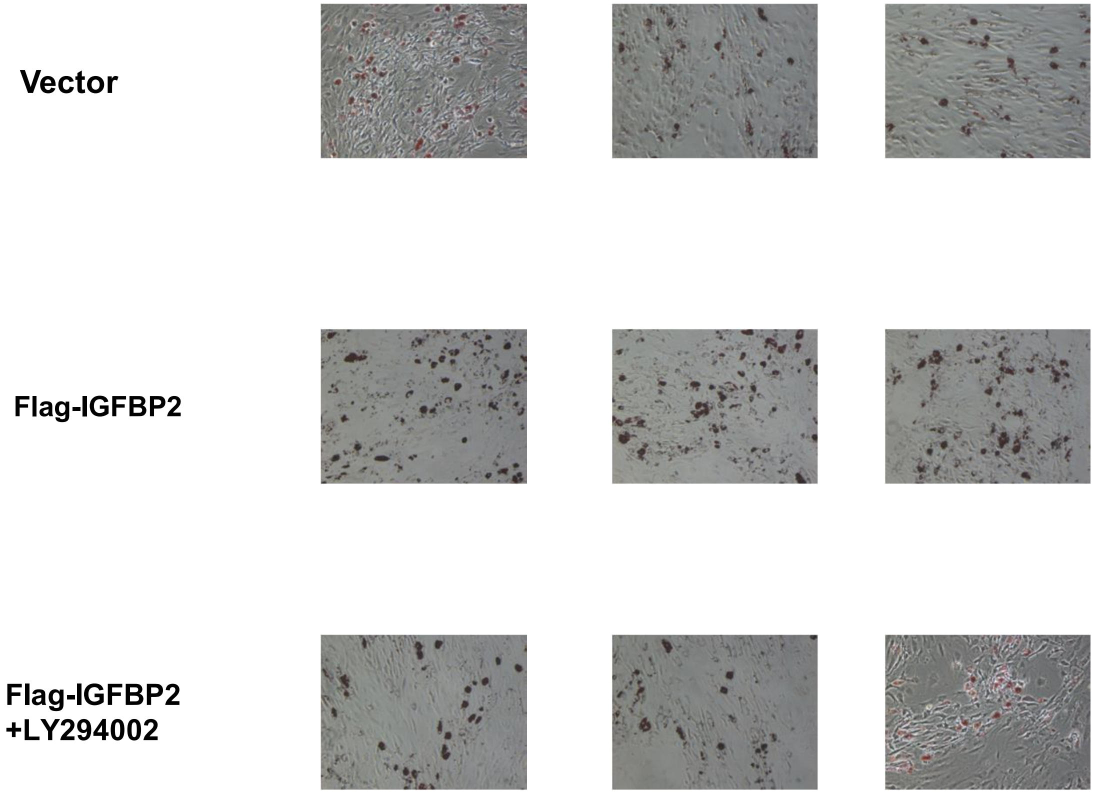

Fig 5C

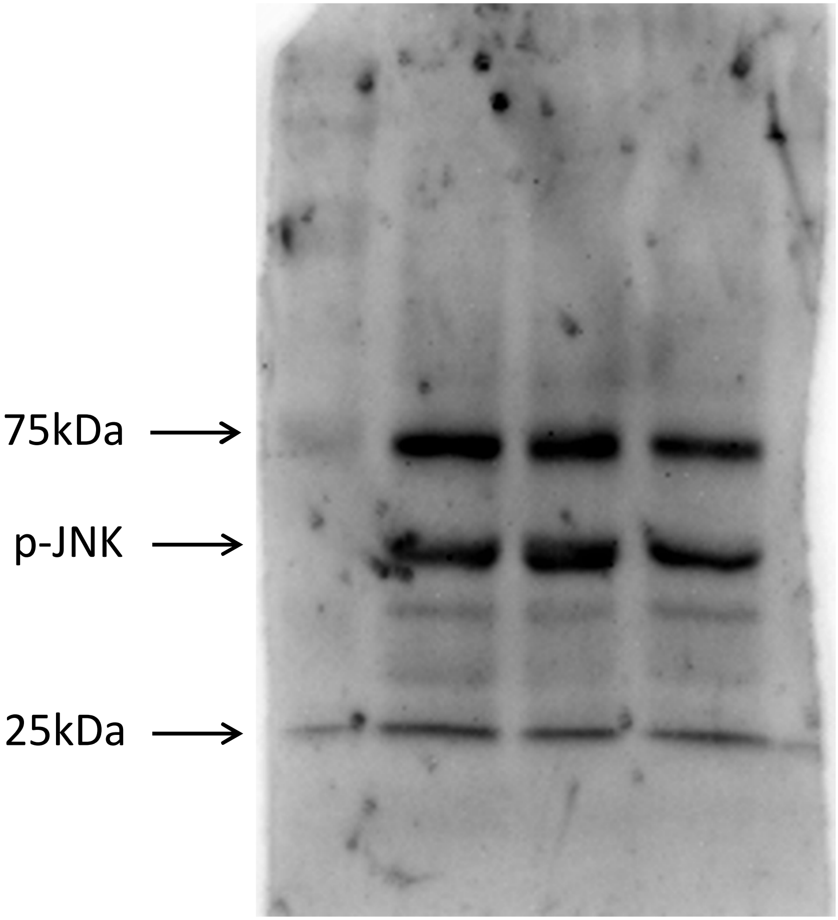

Fig 6A p-JNK

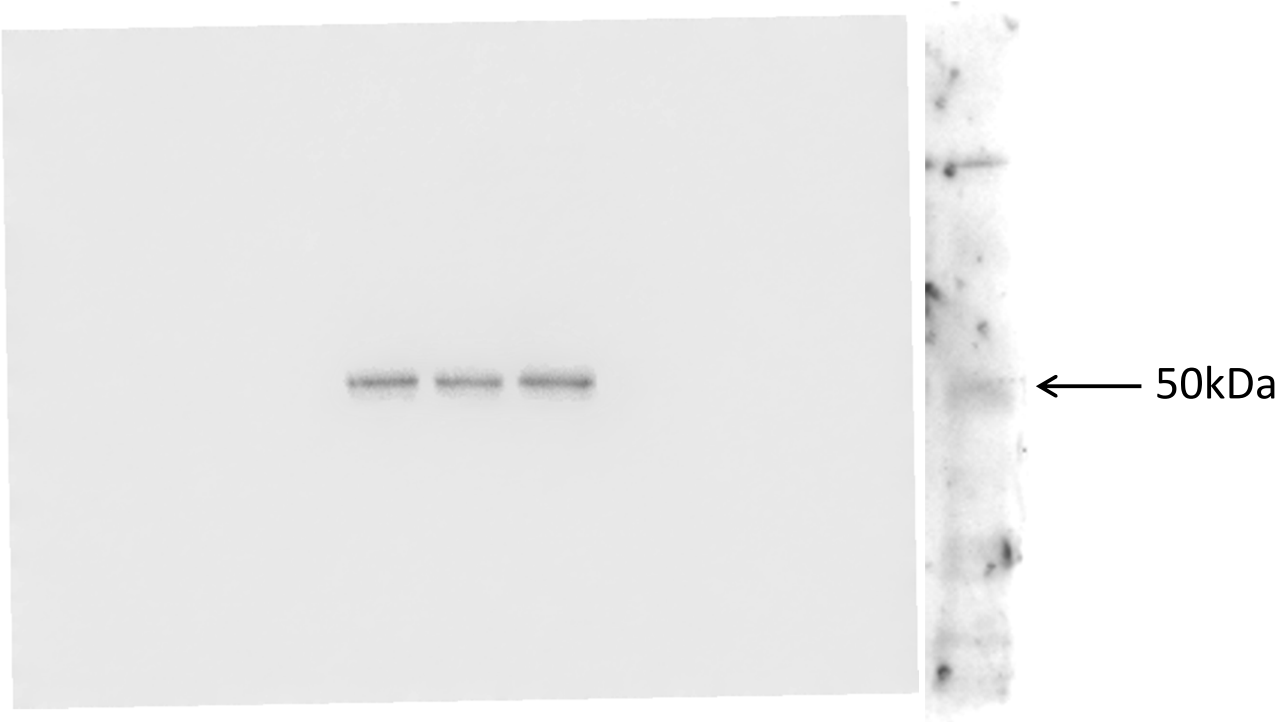

Fig 6A JNK

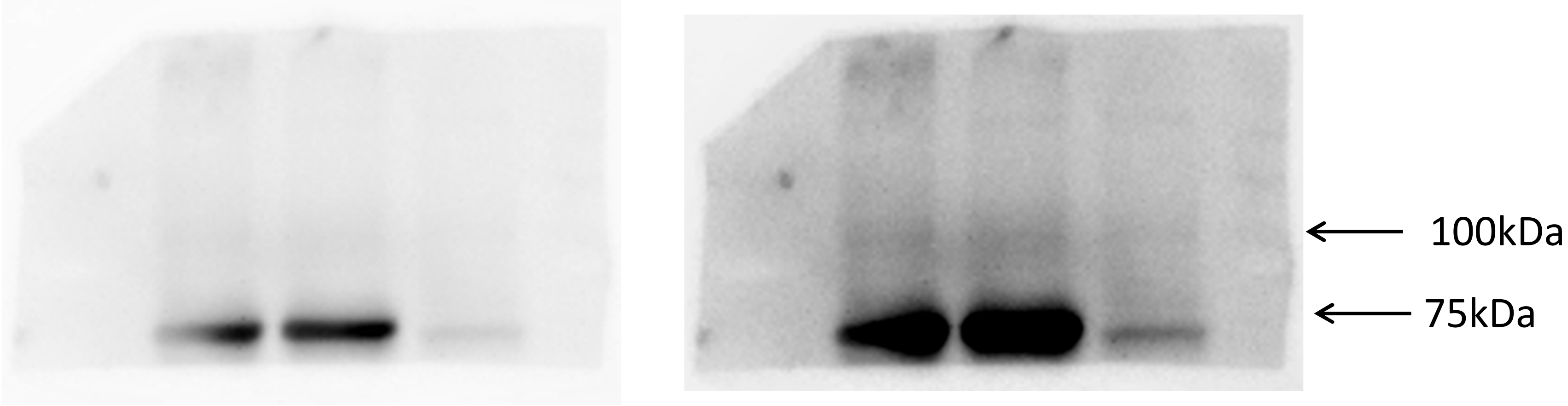

Fig 6A p-Akt

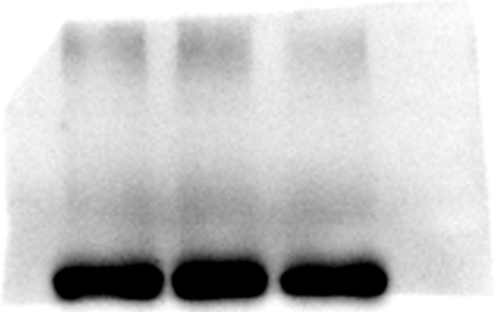

Fig 6A Akt

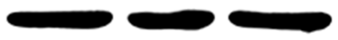

Fig 6A GAPDH

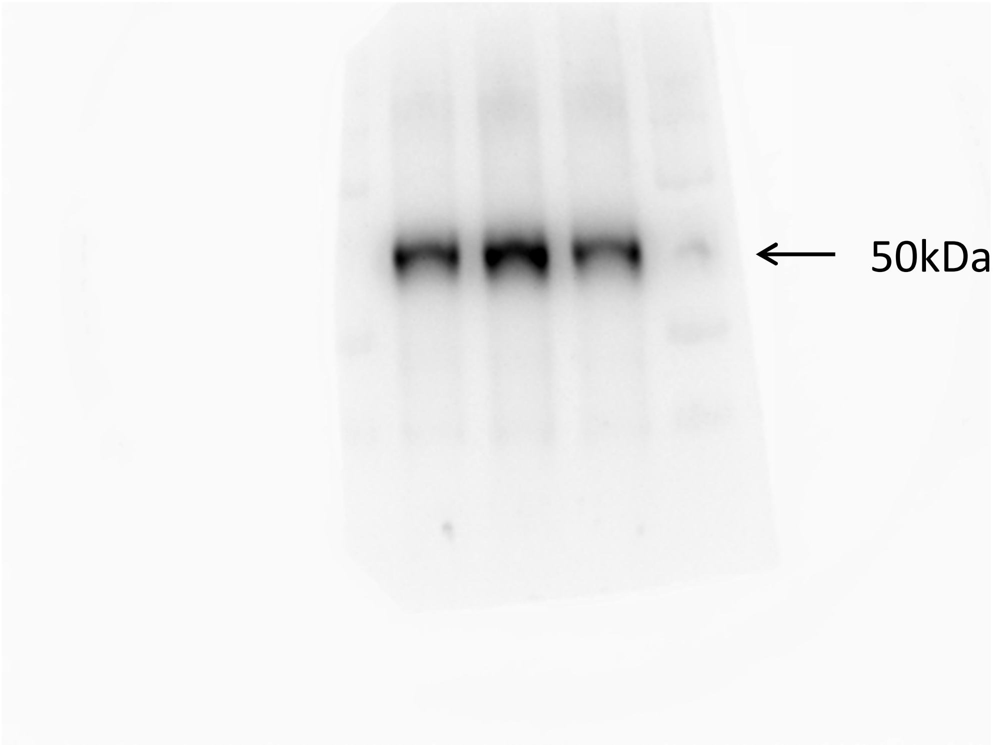

Fig 6C p-Akt

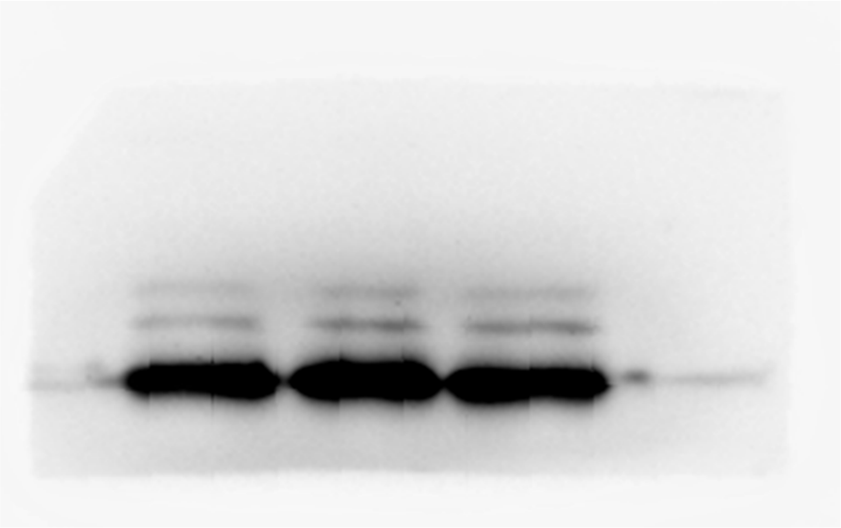

Fig 6C Akt

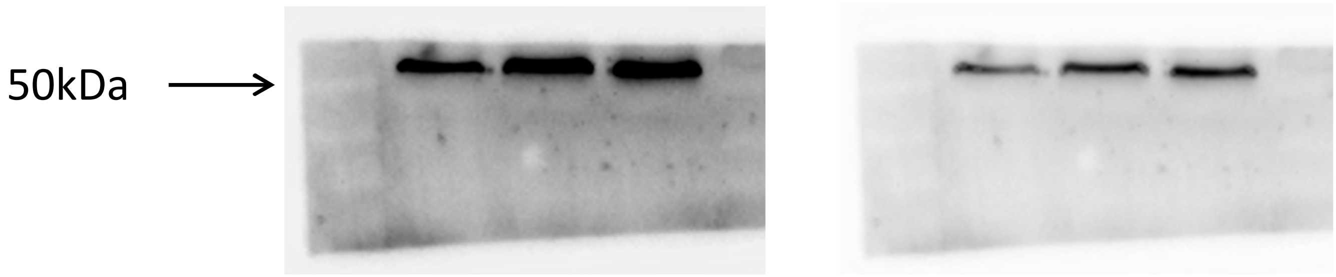

Fig 6C p-JNK

50kDa

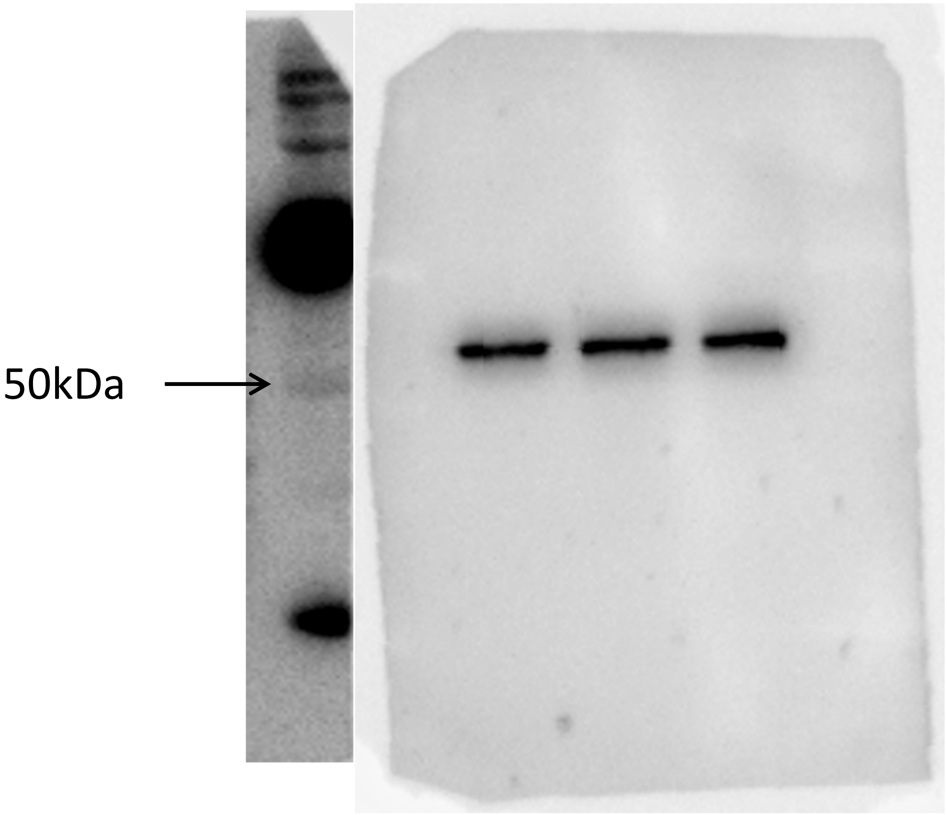

Fig 6C JNK

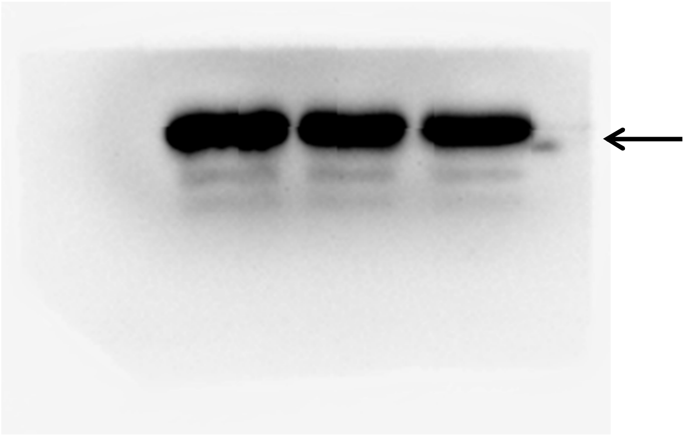

Fig 6C GAPDH

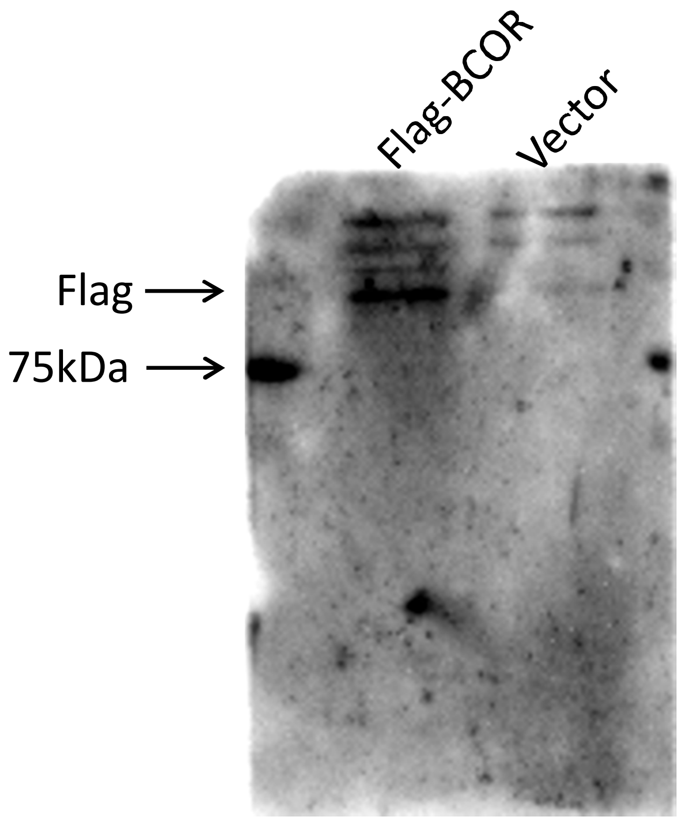

Fig 7A Flag-BCOR

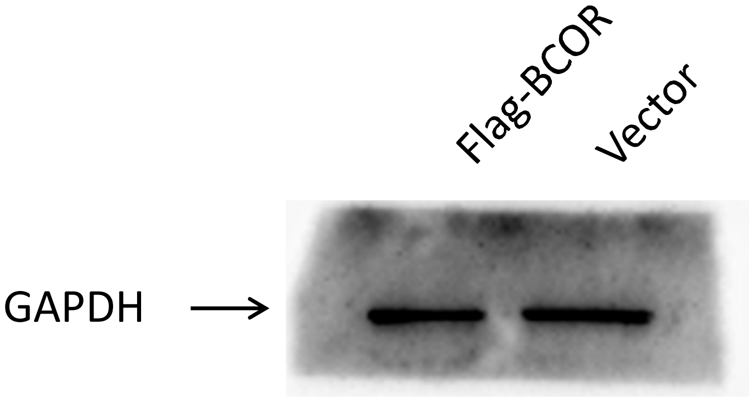

Fig 7A GAPDH

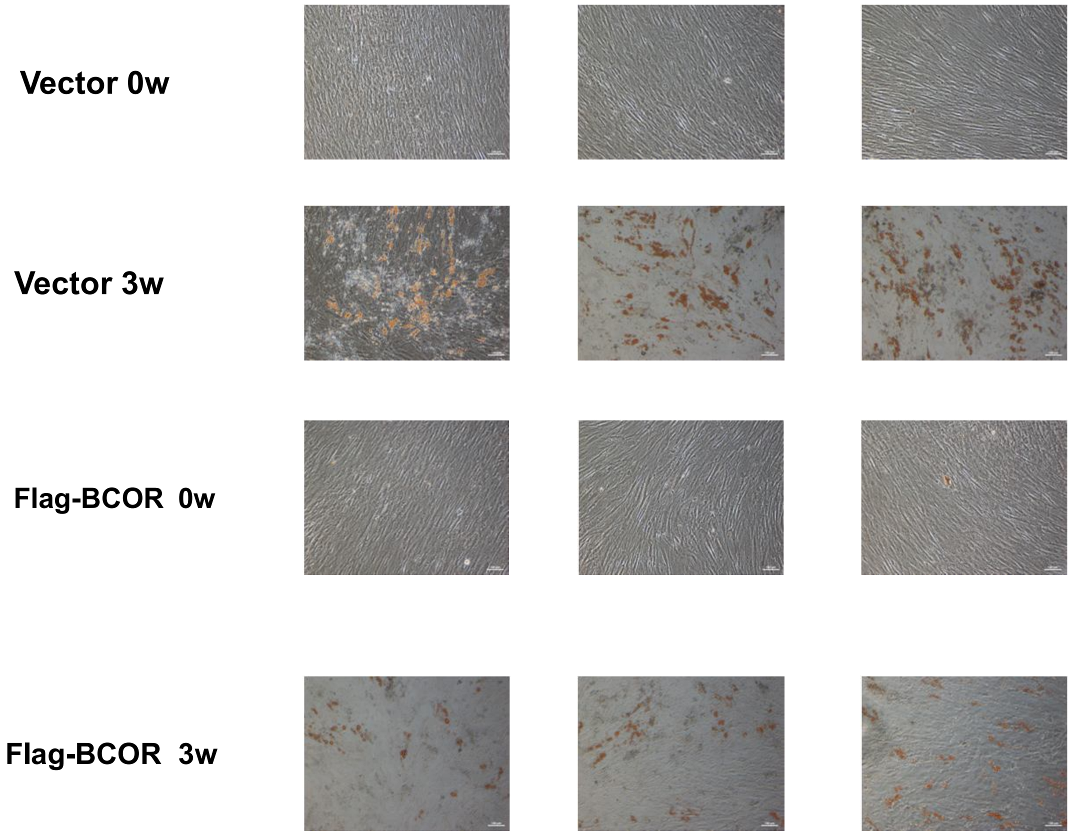

Fig 7C

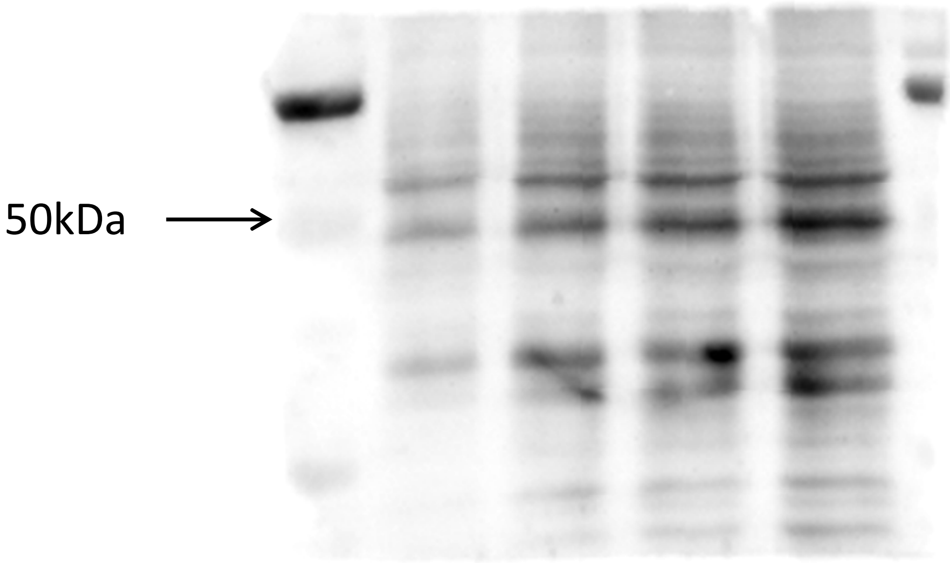

Sfig 2A P-JNK

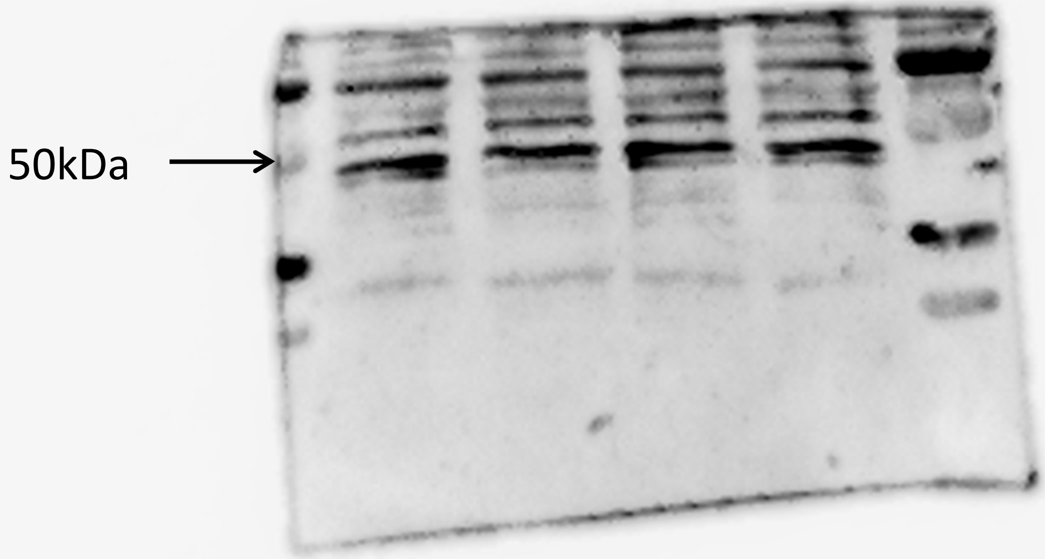

Sfig 2A JNK

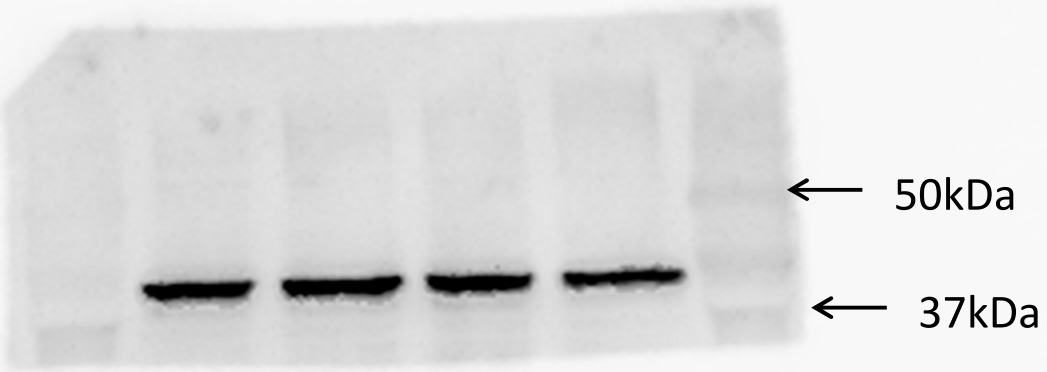

Sfig 2A GAPDH

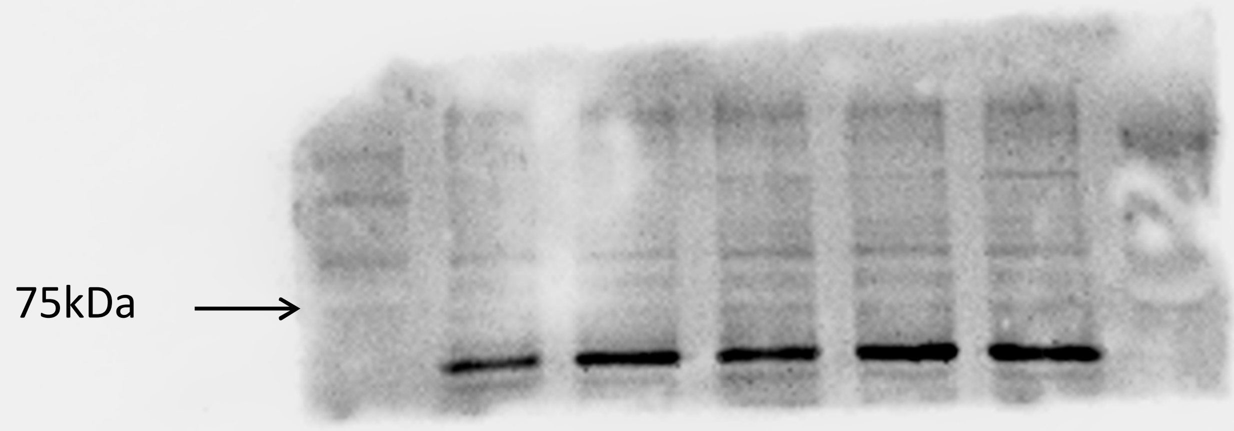

Sfig 2C p-Akt

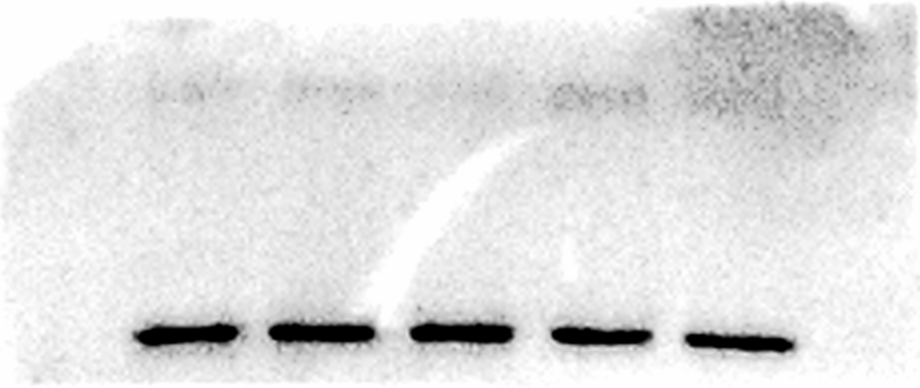

Sfig 2C Akt

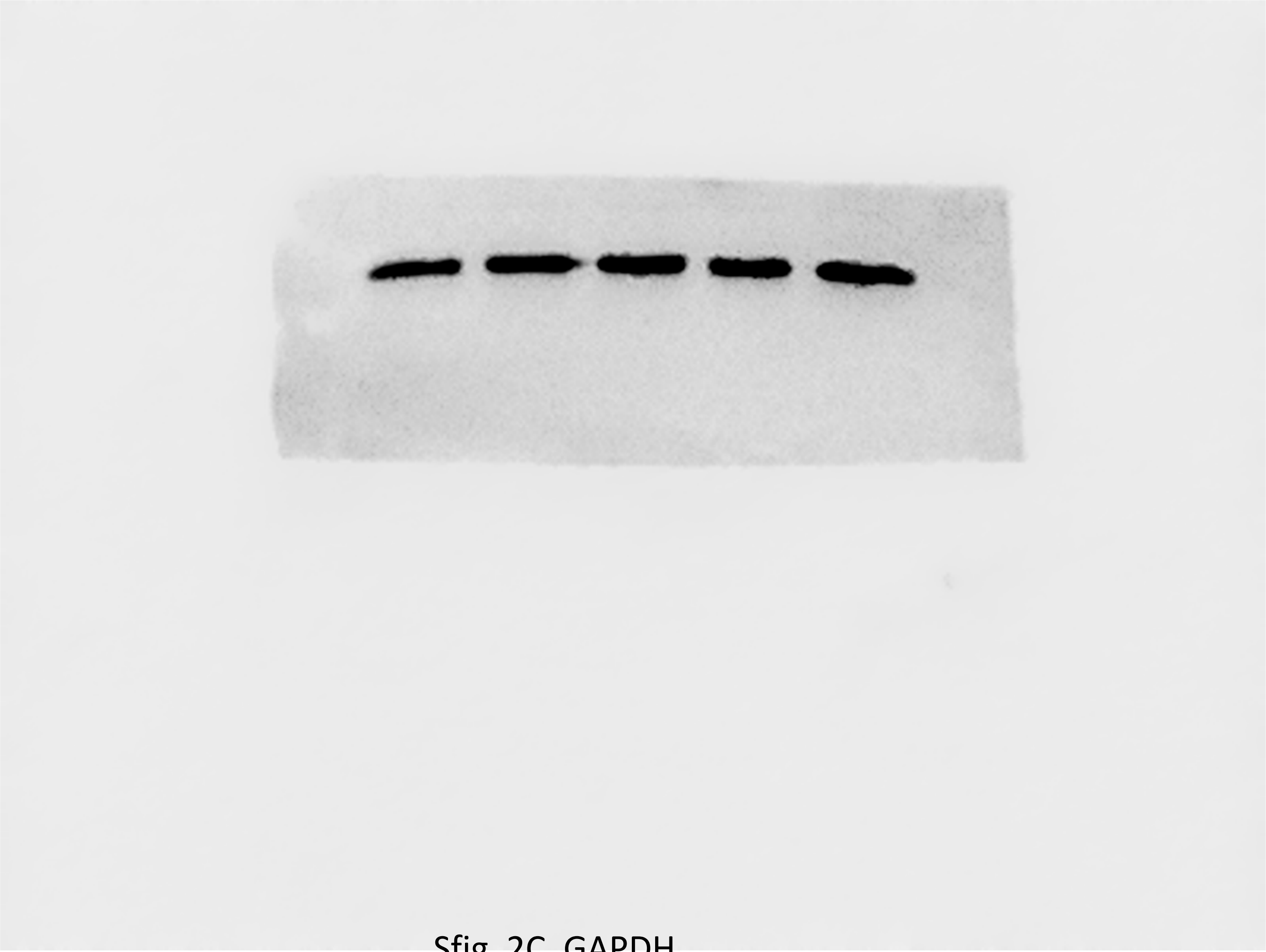

Sfig 2C GAPDH

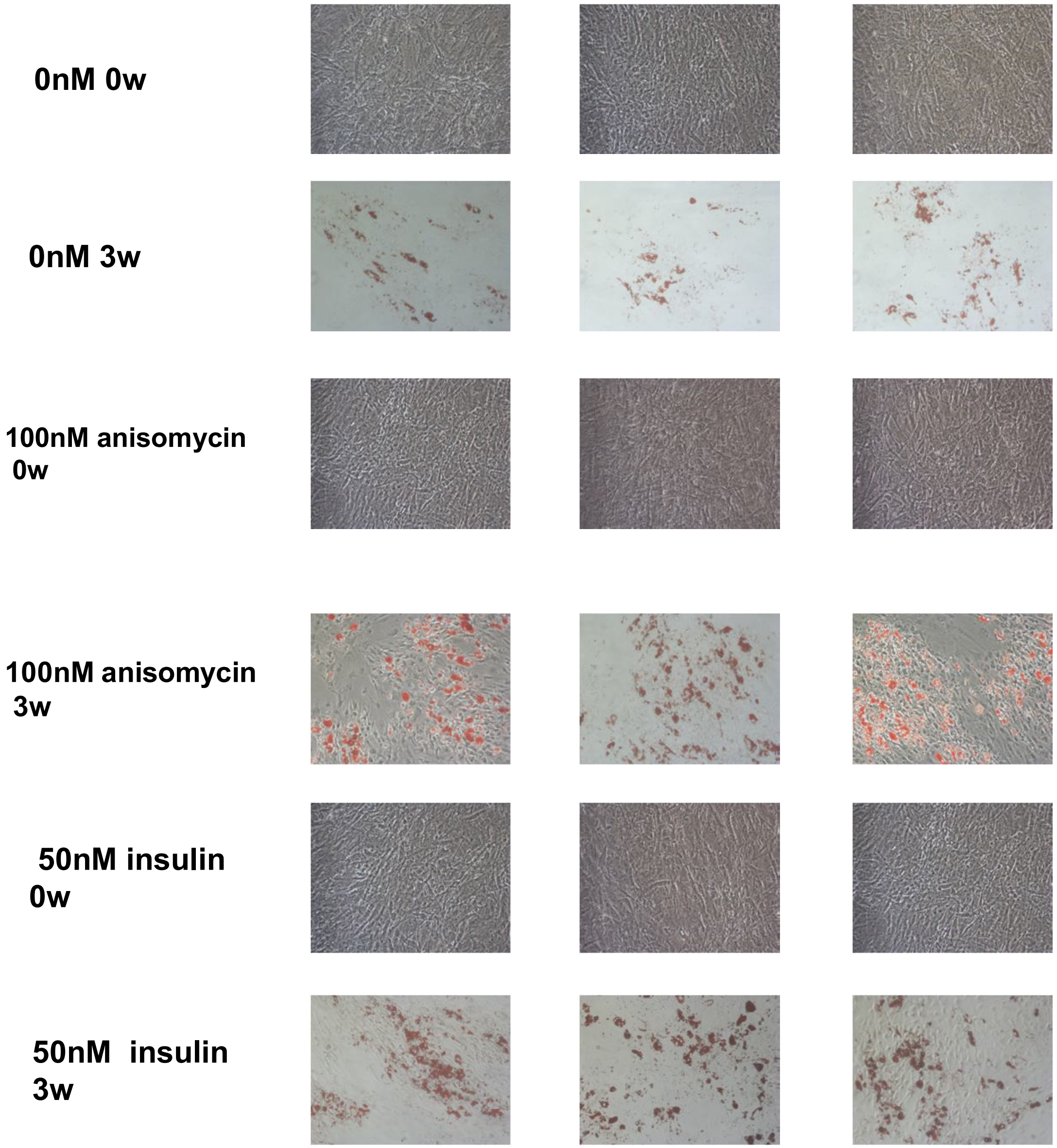
0nM 0w
0nM 3w
100nM anisomycin 0w
100nM anisomycin 3w
50nM insulin 0w
50nM insulin 3w

Sfig 2E

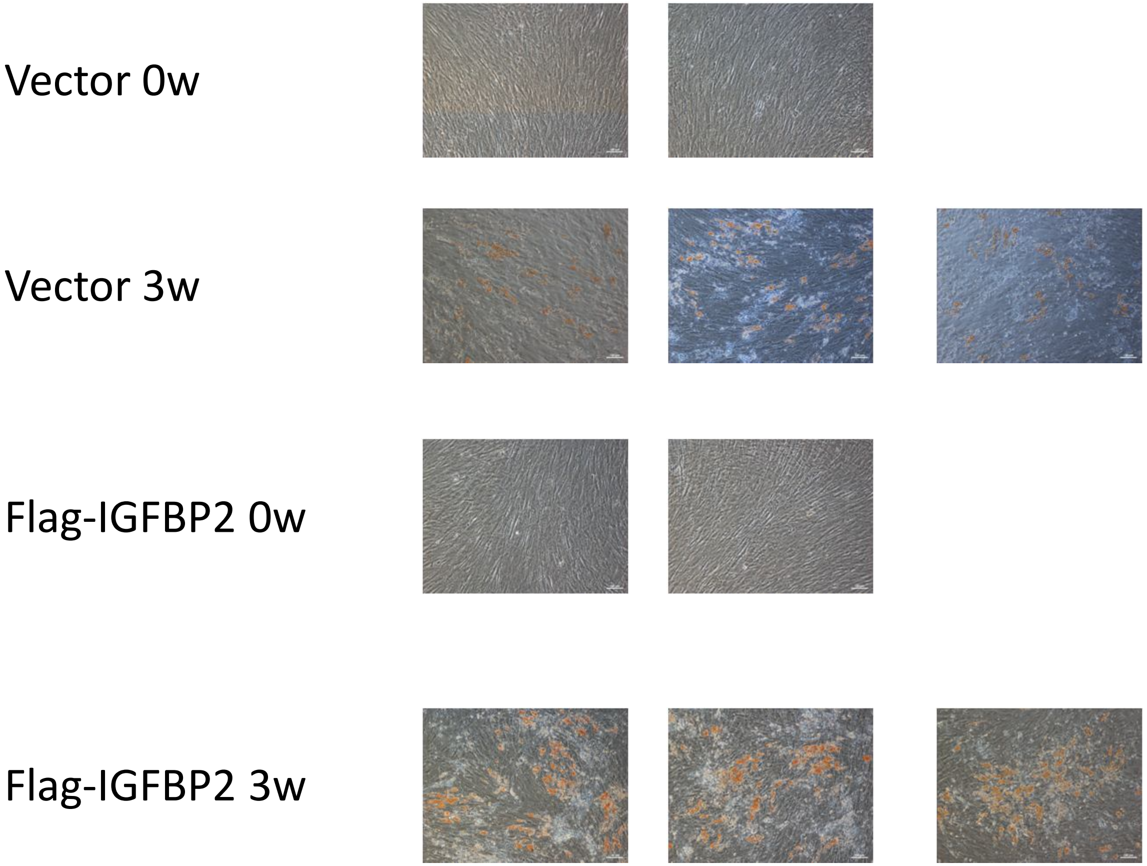

Sfig 1B
